# CRISPR-mediated HDAC2 disruption identifies two distinct classes of target genes in human cells

**DOI:** 10.1371/journal.pone.0185627

**Published:** 2017-10-05

**Authors:** Priyanka Somanath, Rachel Herndon Klein, Paul S. Knoepfler

**Affiliations:** 1 Department of Cell Biology and Human Anatomy, University of California, Davis, Davis, CA, United States of America; 2 Institute of Pediatric Regenerative Medicine, Shriners Hospital For Children Northern California, Sacramento, CA, United States of America; Università degli Studi di Milano, ITALY

## Abstract

The transcriptional functions of the class I histone deacetylases (HDACs) HDAC1 and HDAC2 are mainly viewed as both repressive and redundant based on murine knockout studies, but they may have additional independent roles and their physiological functions in human cells are not as clearly defined. To address the individual epigenomic functions of HDAC2, here we utilized CRISPR-Cas9 to disrupt *HDAC2* in human cells. We find that while *HDAC2* null cells exhibited signs of cross-regulation between HDAC1 and HDAC2, specific epigenomic phenotypes were still apparent using RNA-seq and ChIP assays. We identified specific targets of HDAC2 repression, and defined a novel class of genes that are actively expressed in a partially HDAC2-dependent manner. While HDAC2 was required for the recruitment of HDAC1 to repressed HDAC2-gene targets, HDAC2 was dispensable for HDAC1 binding to HDAC2-activated targets, supporting the notion of distinct classes of targets. Both active and repressed classes of gene targets demonstrated enhanced histone acetylation and methylation in HDAC2-null cells. Binding of the HDAC1/2-associated SIN3A corepressor was altered at most HDAC2-targets, but without a clear pattern. Overall, our study defines two classes of HDAC2 targets in human cells, with a dependence of HDAC1 on HDAC2 at one class of targets, and distinguishes unique functions for HDAC2.

## Introduction

Epigenetic regulation of gene expression is mediated by chromatin modifying complexes that orchestrate transcriptional activation or repression. For instance, the dynamic addition and removal of acetyl groups on specific lysine residues of the amino-terminal tails of histones within gene regulatory domains by specific protein complexes is a key epigenomic mechanism of transcriptional control. Net histone acetylation within chromatin regions is mediated by the interplay of histone acetyl transferases (HATs), which mediate the acetylation of lysines on histones to generally promote a transcriptionally active chromatin state, and the HDACs, which catalyze the removal of acetyl groups that is largely thought to induce a more repressive chromatin state. The Class I HDACs including HDAC1, HDAC2, HDAC3 and HDAC8, are widely expressed and are the most thoroughly studied of the HDAC families [1,2]. HDAC1 and HDAC2 are most homologous to each other. Lacking known target specificity themselves, HDAC1 and HDAC2 together function as the catalytic core of three major co-repressor complexes: SIN3A/B, NuRD (nucleosome remodeling and deacetylation), and CoREST (co-repressor for element-1 silencing transcription factor) [3–6]. In turn, these HDAC1 and HDAC2-containing co-repressor complexes are recruited by specific transcription factors through DNA recognition motifs that bring HDACs to specific nucleosomal domains where they can act on histone substrates [7]. HATs are often recruited by coactivators and other transcription factors to the same domains, establishing an overall machinery that regulates transcription via histone acetylation. One of the best examples of this HDAC-HAT dynamic is the antagonism between MYC-MAX dimer recruitment of HATs to many of the same E-box containing regions targeted by MXD-MAX repressive dimers that recruit SIN3-HDAC1-HDAC2 complexes [8].

HDAC1 and HDAC2 share a high degree of amino acid identity (83% and 86% in mouse and human, respectively), and form heterodimers with enhanced enzymatic activity [9]. Depending on the cellular context, HDAC1 and HDAC2 can either exist in predominantly heterodimer form in both normal and cancer cells [10], function independently, or potentially act as homodimers in other cellular contexts such as in mouse fibroblasts [11]. Genome-wide mapping of HDAC1 and HDAC2 binding has also revealed locations where they bind independently from each other [12]. Although such studies have suggested that the two enzymes may have distinct roles in addition to their integrated function as dimers, previous reports have largely shown that only double knockout of both HDAC1/2 generates a consistently pronounced phenotype, depicting them as either largely functionally redundant or robustly compensatory [13–15]. However, there appear to be some clearly distinct functions. For example, only HDAC1 is essential for mouse germline cell function and for determining specific differentiation decisions of mouse embryonic stem cells (ESCs) [16]. A common phenotype of HDAC1/2 double knockouts is a reduction in cell proliferation [13,14,17]. Several small-molecule inhibitors of HDACs are available that have made loss-of-function of HDACs in human cells possible; however, most inhibitors do not allow for distinguishing the specific roles of class I HDACs such as HDAC1 versus HDAC2 [10]. Therefore, it remains unclear as to whether HDAC1 and HDAC2 have entirely independent repressive functions, gene targets, or impacts on cellular functions. As HDAC2 is aberrantly expressed in several types of cancer including gastric, colorectal, prostate and Hodgkin’s Lymphoma [18–20], distinguishing the precise functions of HDAC2 remains important for understanding epigenetic mechanisms of human diseases, as the contribution of HDAC2 to tumorigenesis may involve functions that are independent of HDAC1. Additionally, HDAC2 may have unique functions as its knockdown has been found to promote the maturation of induced pluripotent stem cells (IPSCs) [21].

Notably, disruption of HDAC1, HDAC2, or both can in some instances lead to decreased gene expression. For example, recent chromatin immunoprecipitation (ChIP) and transcriptomic analysis from HDAC inhibition in yeast cells or HDAC1 knockout studies in mice have shown a significant portion of downregulated genes signifying that these proteins may also work to functionally activate genes in the physiological state [12,22,23]. However, the mechanisms by which HDACs contribute to gene activation remain unclear and in some cases could be indirect. Interestingly, genome-wide mapping of class-1 HDAC localization linked some instances of direct binding of HDAC1 and HDAC2 with gene activation as they and their associated proteins bound at some transcriptionally active loci with acetylated histones in human and mouse cells [12,24]. Those findings suggest HDACs help reset active chromatin domains potentially to prevent inappropriate re-initiation of transcription that subsequently allows for reactivation through inhibition of RNA polymerase II activity. However, the unique targets which HDAC1 or HDAC2 activate have not been clearly established, and an interesting open question remains of whether HDAC1 or HDAC2 can also function within distinct protein complexes from their established major co-repressor complexes to mediate this activation-associated function.

To address HDAC2 function in human cells, here we used CRISPR-Cas9 targeting of *HDAC2* to produce 293FT cells with *HDAC2* loss-of-function. CRISPR-mediated production of disruptive Indels in *HDAC2* successfully lowered HDAC2 protein levels to barely detectable, near background levels indicative of effectively null cells. Loss of HDAC2 does not lead to consistent compensatory increases in HDAC1 or HDAC3, but HDAC1 and HDAC2 are tightly functionally interconnected. Knockout of *HDAC2* leads to both increased and decreased expression of specific genes. HDAC2 is most often required for the recruitment of HDAC1 to repressed target genes bound by HDAC2. While HDAC2 contributes to activation of some direct gene targets, its disruption leads to either no change or increased HDAC1 binding at these gene targets suggesting a compensatory function specifically at this class of targets. In both classes of targets, we find that SIN3 binding is variably affected by loss of HDAC2 at individual genes. Overall, our data support a model of two separate mechanisms of HDAC2 function as either contributing to gene repression or activation with distinct roles for HDAC1 in each case.

## Materials and methods

### Cell culture and transfections

HEK293FT cells were cultured in DMEM supplemented with 10% FBS (HyClone) and 1% L-glutamine. Transfections were conducted with Roche X-tremeGENE HP DNA transfection reagent (Roche). Media was changed 24-hours post transfection.

### CRISPR-Cas9 targeting of *HDAC2*

Guide RNAs targeting the first exon of HDAC2 were designed using the web tool http://crispr.mit.edu. Oligos for the guide targeting the sequence 5’*CCC****ATG****GCGTACAGTCAAGGAGG* (first ATG bolded) were annealed and ligated into the Bbs1-digested pSpCas9(BB)-2A-Puro vector (Addgene) as described [25]. Integration into the plasmid was confirmed by sequencing and guide RNA-encoding plasmids were transfected as described above into 293FT cells. 48-hour post-transfection, cells were trypsinized to single cells and puromycin (1.5 ug/mL) was used to select for transfected cells for approximately 3 weeks to generate stable clonal lines from single cells. Individual colonies were harvested and genomic DNA isolated from each using the QuickExtract DNA Extraction Solution (Epicentre). We PCR amplified the *HDAC2* gene targeted region using the primers: Forward 5’-*CTAACCTCGAGCCCGAAACG*-3’ and Reverse *5’-CTCGTTCTAACTGTGCCGGG*-3’. The resulting PCR products were cloned into the pCR4-TOPO vector using the TOPO-TA Cloning Kit (Invitrogen) according to the manufacturer’s instructions and DNA from at least 6 colonies were sequenced per clonal line to screen for and characterize any Indels present in each. The top four possible off-target regions predicted by the website tool were also sequenced by PCR amplification of genomic DNA from each clonal line. Primer sequences for off-target region amplification are listed in S1 Table. Criteria for off-target region selection is listed in S2 Table.

### RNA-seq

Three plates each of wild-type (WT) cells or HDAC2-null lines #5, #14, or #15 were grown to 60 to 70% confluence and total RNA was isolated from each clonal line separately using the Nucleospin RNA Kit (Macherey-Nagel, Clontech), with DNase treatment 5 μg RNA was submitted to the UC Davis DNA Technologies Core for library preparation utilizing the KAPA stranded mRNA-seq kit (KK8421, KAPA Biosystems), and sequenced as single reads (SR50) on the Illumina HiSeq3000 platform. WT and each clonal line were Poly-A enriched prior to sequencing. RNA-seq reads were aligned using Tophat [26,27] and quality was inspected with RSeQC [28] (S3 Table). The sequence-based and mapping-based duplication rates were calculated with RSeQC read_duplication.py. Two of the twelve replicates, one WT and one from line 15, were found to be distant outliers and removed from further analysis (S1 Fig). Although PCA analysis indicated substantial internal variability between sample groups themselves, with the two total major outliers removed, the control replicate samples tended to cluster together and away from the HDAC2-CRISPR targeted replicates (S1 Fig). After removal of the two outliers, differential expression between WT (two replicates) and the three HDAC2 depleted clonal lines each grown separately (eight replicates) was determined as ten total biological replicates with the EdgeR Bioconductor package (version 3.3.2) using the classic method (estimating the quantile-adjusted conditional maximum likelihood common and tagwise dispersions and identifying differentially expressed genes with the exact test) [29,30]. The FDR method used was Benjamini-Hochberg. Differentially expressed genes were defined with a FDR cutoff of 0.3. The significance of the overlap of affected genes in the three clonal lines was calculated using the SuperExactTest R package (PMC4658477) [31]. GOseq was used for gene ontology analysis of differentially expressed genes [32]. Data on differential gene expression in *HDAC2*-null lines relative to WT cells identified through EdgeR analysis of RNA-seq data were also compared to differential gene expression data produced using Cufflinks analysis of the same RNA-seq data for validation.

### qPCR

RNA was reverse transcribed using the iScript cDNA Synthesis Kit (BioRad) and qPCR assays were performed in triplicate technical replicates utilizing Absolute Blue qPCR SYBR Green Master Mix (Fisher Scientific) and 5 μL cDNA on Roche Light Cycler 480 at the following conditions: 95°C for 15 seconds, 60°C for 30 seconds and 72°C for 30 seconds for 45 cycles. Expression was normalized to *GAPDH* endogenous control and analyzed via the double ΔCT method. Student’s t-test was performed to assess statistical significance. Primers are listed in S4 Table.

### ChIP assays

ChIP assays were performed as described [33,34]. Briefly, 2.5 million cells were crosslinked with 37% w/v formaldehyde, quenched with 2.5M glycine, washed 3 times with ice cold PBS, and sonicated in a Bioruptor (Diagenode). The following primary antibodies were utilized for incubation of crosslinked chromatin overnight: HDAC1 (Abcam #31263, 3μg), HDAC2 (Abcam #12169, 3μg) pan-H4ac (Upstate #06–866, 5μg), H3K9Ac (Abcam #12179, 5μg), H3K9me3 (Abcam #8898, 5μg), SIN3A (Abcam #3479, 5μg) and as a negative control IgG Rabbit (Cell Signaling Technologies #2729S, 3 or 5μg), IgG Rabbit (Santa Cruz #2027, 3 or 5μg), or IgG Mouse (Santa Cruz #2025, 3 or 5μg,). Protein A/G magnetic beads (Pierce #88802) were added at 50 μL per ChIP sample. ChIP-qPCR primers were designed using Primer3 web tool and are listed in S5 Table.

### Western blotting

Nuclear lysates were prepared according to Abcam Nuclear Fractionation protocol. Briefly, cells were scraped into ice-cold PBS and resuspended into Buffer A (10mM Hepes, 1.5mM MgCl2, 10mM KCl, 0.5 mM DTT, pH 7.9) and incubated for 10 minutes. Pellets were resuspended in Buffer B (5mM HEPES, 1.5 mM MgCl2, 0.2 mM EDTA, 0.5 mM DTT, 26% glycerol v/v, 300 mM NaCl, pH 7.9) for 20 minutes, centrifuged, and nuclear fraction (supernatant) was used for analysis. Protease inhibitor cocktail tablet (cOmplete Mini EDTA-free, Roche) was added to all buffers. Histone acid extracts were prepared as described [35]. Protein samples were electrophoresed on NuPAGE 4–12% Bis-Tris gels (Invitrogen) as described [36] and transferred to polyvinylidene fluoride (PVDF) membranes. Membranes were blocked in Blok-FL Fluorescent blocker (Millipore) and incubated with primary antibodies overnight: HDAC1 (1:1000), HDAC2 (1:2000), HDAC3 (Abcam #7030, 1:1000), pan-H4ac (1:2000), H3K9ac (1:2000), H3K9Me3 (Upstate #07–523,1:2000). Blots were incubated with goat anti-mouse (1:10,000 dilution IR Dye 680RD, LiCOR) or goat anti-rabbit secondary antibodies (1:10,000 dilution IR Dye 800CW, LiCOR) for one hour at room temperature and imaged on an Odyssey Imaging system (LiCOR). Blots were internally normalized with beta-actin (Sigma #A1978, 1:5000). All Western blots were done with three biological replicates and statistical significance assessed through unpaired Student’s t-test.

## Results

### CRISPR-Cas9 targeting of *HDAC2* generates clonal lines that have disrupted *HDAC2*, and altered HDAC3, but not HDAC1 protein levels

**Fig 1:**
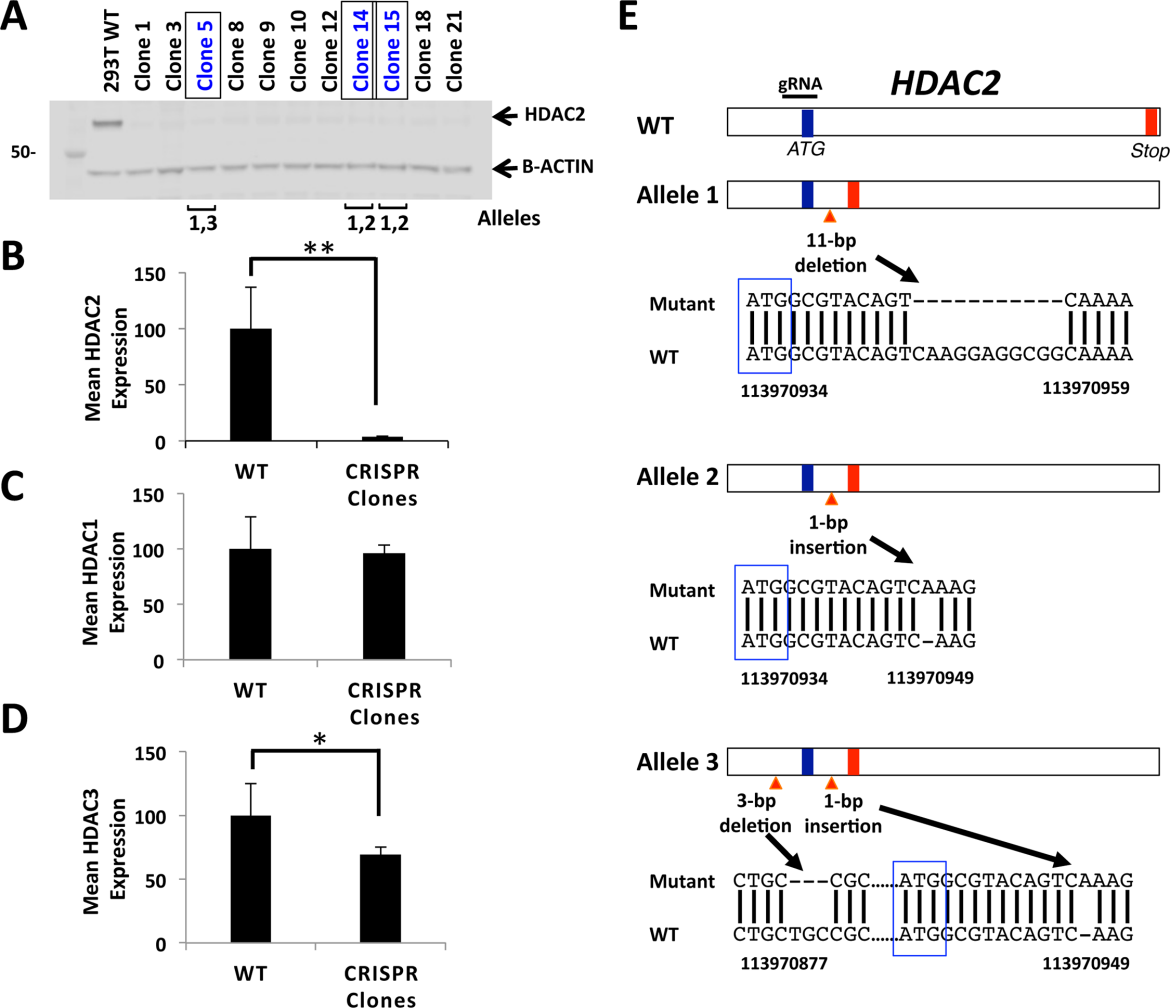
CRISPR-Cas9 disruption of *HDAC2* in human cells. (**A)** Nuclear lysates of a panel of *HDAC2* targeted independent clonal lines generated through transfection of 293FT cells with CRISPR-Cas9 guide-RNA expressing plasmids targeting the first ‘ATG’ of *HDAC2* were analyzed by Western blotting, demonstrating a complete or near complete loss of HDAC2 protein. (**B)** A larger panel of 21 *HDAC2*-targeted clonal lines demonstrated an overall mean 97% reduction in HDAC2 protein levels compared to WT HDAC2 protein (p<0.001). See S2A–S2C Fig for additional individual clone HDAC2 protein level data. **(C)** Mean HDAC1 protein levels in a panel of 13 *HDAC2* null clonal lines demonstrated no significant change compared to HDAC1 protein levels in WT control. See also S2D and S2E Fig. **(D)** Mean HDAC3 protein levels for a panel of 13 *HDAC2* null clonal lines demonstrated an overall mean 31% reduction of HDAC3 protein levels compared to WT cells (p<0.05). See also S2F and S2G Fig. All protein levels were normalized to B-actin. **(E)** Genomic DNA was isolated from three independent candidate gene edited clonal lines, PCR-amplified *for HDAC2*, and sequenced to detect and characterize any Indels present in each line. Three distinct alleles were identified. Allele 1 harbors a 11-bp deletion 3’ of the first ATG, Allele 2 has a 1-bp insertion 3’ of the first ATG, and Allele 3 has a 3-bp deletion 5’ of the first ATG and a 1-bp insertion 3’ of the first ATG. All three alleles result in a premature stop codon (both WT and Indel-induced stop codons are indicated as red vertical lines, while start codons are indicated by blue vertical lines). The three clonal lines of focus for this study overall are indicated by brackets in panel (A): clone 5 has alleles 1 and 3, while clonal lines 14 and 15 have alleles 2 and 3.

In order to study the transcriptional outcomes of HDAC2-depletion in a human cellular context, we chose the highly tractable 293FT cells, which are easily transfected, selected and screened, to generate a panel of HDAC2 loss-of-function human lines via CRISPR-Cas9. We employed a guide RNA (gRNA) sequence designed to target the first *ATG* of the *HDAC2* gene with low probability of off-target binding. To test the predicted CRISPR-mediated knockout of *HDAC2*, HDAC2 protein levels were examined in the overall panel of clonal lines and each exhibited nearly undetectable HDAC2 protein levels compared to the parental WT control line (mean p<0.001, Fig 1A and 1B and S2A–S2C Fig). Since the mean reduction in HDAC2 band intensity was >96% and only putative *HDAC2* null alleles (see next section) are present in these clonal lines, it is unclear whether the very faint residual protein band present in the targeted lines at approximately the same size as HDAC2 is actual residual HDAC2 protein present (e.g. potentially small amounts of long-lived, carryover HDAC2 protein protected within dense heterochromatin) or a background band. A panel of 13 HDAC2 loss-of-function clonal lines, was analyzed for HDAC1 and HDAC3 protein levels to determine if there were consistent changes in other class I HDAC family member levels with the knockout of *HDAC2*. We found that HDAC1 protein levels were not significantly changed across the clonal line panel (Fig 1C and S2D and S2E Fig). However, each of the clonal lines examined exhibited a decrease of HDAC3 protein levels by an overall mean of 31% with p<0.05 (Fig 1D and S2F and S2G Fig). While these data correlate with published reports of a decrease in HDAC3 protein levels in *Hdac2*-knockout mouse ESCs [16], the decrease in HDAC3 protein we observed was in nuclear lysates of HDAC2-depleted cells and as HDAC3 is known to shuttle between the nucleus and cytoplasm [1,2], it remains possible that our observation may be due to decreased HDAC3 in the nuclear compartment alone. Overall, these data argue against compensation for loss of HDAC2 by increased HDAC1 or HDAC3 protein levels.

### *HDAC2* targeted clonal lines contain effectively null alleles

Within the panel of HDAC2-disrupted clonal lines, we more fully characterized three clonal lines (5, 14, and 15) including for the presence of Indels through PCR-sequencing of the targeted region in the *HDAC2* gene. Sequencing indicated that all three clonal lines are hetero-allelic (Fig 1E). Two clonal lines (clones 14 and 15) carry an 11-base pair (bp) deletion 3’ of the first coding *ATG* in one allele, and a 1-bp insertion 3’ of the first *ATG* in the second allele. The third clonal line (clone 5) carries the same 11-bp deletion 3’ of the first *ATG*, but in combination with both a 1-bp insertion 3’ to the first *ATG* and a 3-bp deletion 5’ to the first *ATG* in the second allele. Overall, all alleles in clones 5, 14, and 15 have at least one Indel resulting in a frameshift leading to generation of a premature stop codon that is predicted to generate null alleles. Sequencing of the top four computationally predicted off-target regions (intronic regions in the *BMP15*, *PARP2*, *MYL2*, and *IKBKB* genes) for each clonal line determined that no Indels were present (S3 Fig). Overall, we generated and validated three effectively *HDAC2* null 293FT clonal lines using CRISPR-Cas9, without evidence of compensatory increases in HDAC1 protein levels, allowing us to specifically study the functions of HDAC2 via its disruption.

### Evidence of cross-regulation between HDAC1 and HDAC2

**Fig 2:**
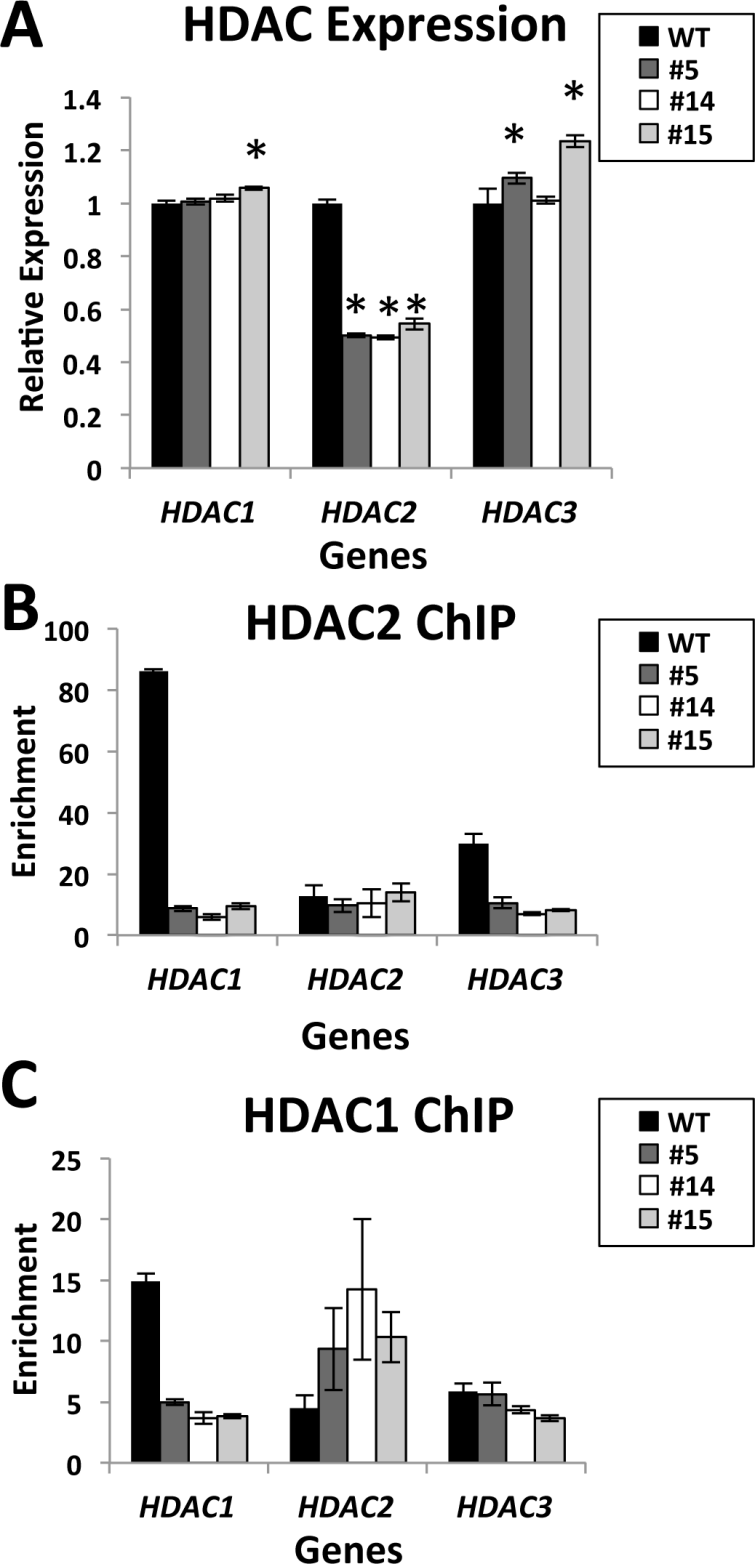
Cross-regulation between Class I HDAC family members is evident in *HDAC2* nulls. **(A)** qPCR measurement of Class I HDAC family expression in control and *HDAC2* null cells was conducted. cDNA from WT cells or *HDAC2* null clonal lines #5, #14 and #15 was subjected to SYBR-green qPCR analysis in triplicate for transcript levels of *HDAC1*, *HDAC2* and *HDAC3*. Gene expression was internally normalized to *GAPDH* and fold enrichment represented relative to WT (* denotes p<0.05 for *HDAC1*, p<0.0001 for *HDAC2*, and p<0.05 for *HDAC3*). Error bars represent standard error of mean (S.E.M.). **(B-C)** Chromatin immunoprecipitation (ChIP) was conducted on crosslinked chromatin of WT, HDAC2 clones #5, #14, and #15 with antibodies to HDAC2 **(B)**, HDAC1 **(C)** or IgG control in each case. SYBR green qPCR analysis was conducted to measure the binding of HDAC2 or HDAC1 at the promoter regions of *HDAC1*, *HDAC2* and *HDAC3*. Enrichment was calculated by normalization to input and IgG control samples and plotted as mean of n = 3 with error bars representing S.E.M.

We quantified the RNA levels of *HDAC1*, *HDAC2*, and *HDAC3* using qPCR analysis to determine the potential effects of *HDAC2* disruption on expression of other class I HDAC family members. CRISPR targeting of *HDAC2* resulted in a mean 50% reduction of *HDAC2* RNA levels in the three clonal lines (Fig 2A), most likely via nonsense-mediated decay. Primers utilized to measure HDAC2 mRNA were designed to target a region not containing indels allowing measurement of mRNA synthesized after CRISPR-mediated HDAC2 disruption (see Methods for additional details on primers). *HDAC1* mRNA levels were not consistently altered in the *HDAC2* null clonal lines. Although HDAC2-disruption was associated with a mean 31% reduction in HDAC3 protein levels (Fig 1D), clonal lines 5 and 15 exhibited a very small (10–20%) but significant increase in *HDAC3* transcript levels.

We also conducted ChIP assays to examine whether the loss of *HDAC2* led to changes indicative of class I HDAC family cross-regulation such as alterations in genomic binding of HDAC2 or HDAC1 at their own or each other’s genes. As predicted, the ChIP assays demonstrated that *HDAC2* disruption resulted in decreased binding of HDAC2 at *HDAC1* and *HDAC3* compared to WT cells, but no change was observed for HDAC2 protein binding at its own gene *HDAC2* (Fig 2B). However, since the level of HDAC2 binding at *HDAC2* was minimal in WT cells, we attributed this very low level to be background binding signal of the ChIP assay. All three *HDAC2*-null lines also exhibited increased HDAC1 binding at *HDAC2*, along with decreased HDAC1 binding at its own gene *HDAC1* in the null cells compared to WT cells (Fig 2C). Collectively, these results support cross-regulation within the class I HDAC family as they indicate that the loss of *HDAC2* affects other HDAC family members by altering HDAC levels or perturbing direct HDAC genomic binding to *HDAC* genes.

### Effects of loss of HDAC2 on the transcriptome

**Fig 3:**
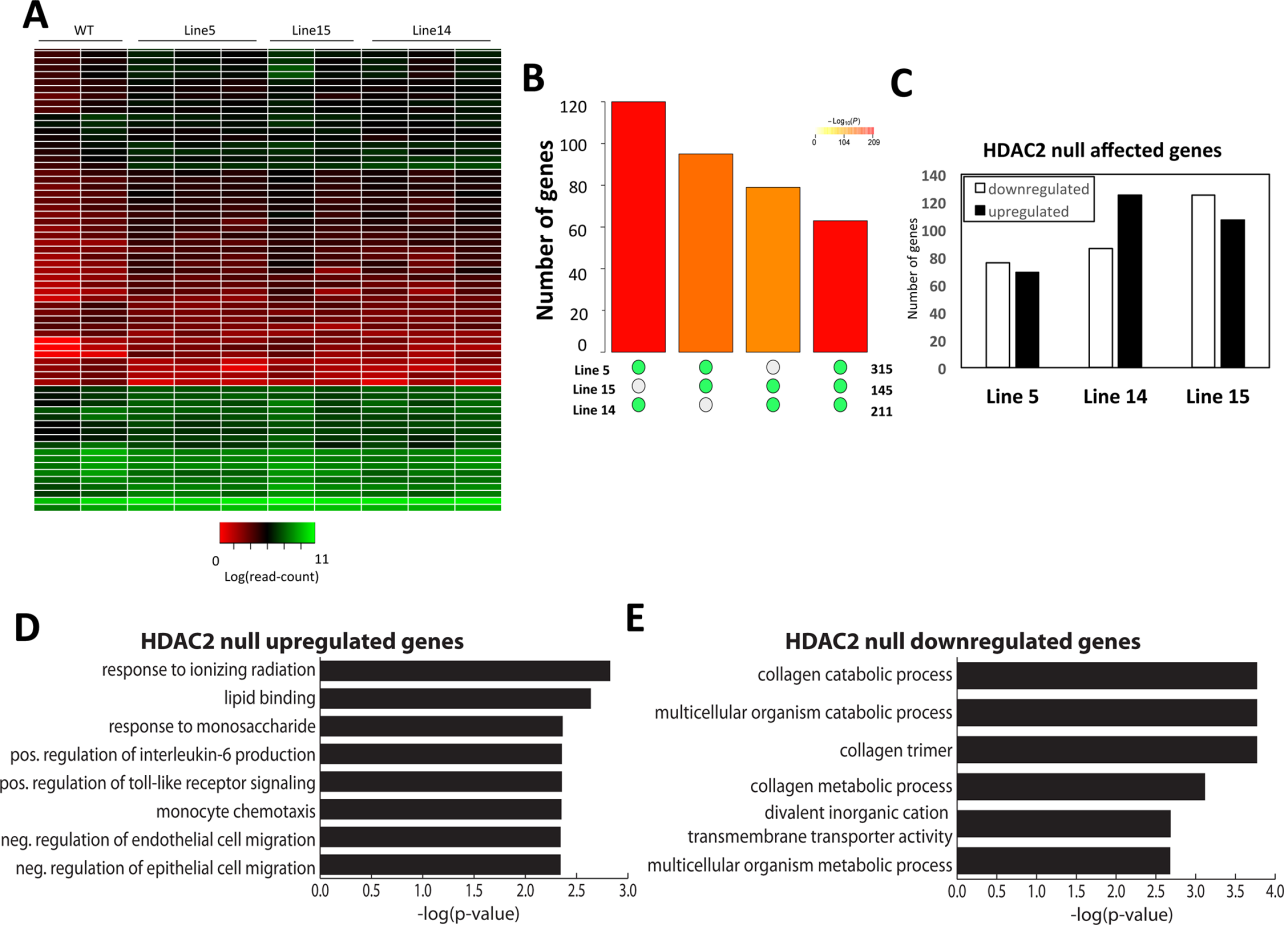
RNA-seq analysis of HDAC2 affected genes. RNA-seq was conducted on control and *HDAC2* null clonal lines to assess transcriptomic effects. **(A)** Heatmap plot of log normalized counts for significantly upregulated and downregulated genes found in the three lines of *HDAC2* nulls compared to WT cells (p<0.01). **(B)** Plot of overlap in transcriptomic changes between each of the three cell lines. The Y axis plots the number of genes that overlap for each comparison, with the color of the bar indicating the p-value, and the different gene set comparisons are indicated on the X axis by green circles. **(C)** Proportions of downregulated and upregulated genes in each *HDAC2* null cell line indicate approximately equal numbers of differentially expressed genes. **(D)** Gene ontology analysis of genes upregulated by *HDAC2* disruption. **(E)** Gene ontology analysis of genes downregulated by HDAC2 disruption.

In order to examine differential expression of the transcriptome between *HDAC2*-null and wild-type (WT) 293FT cell lines, total RNA was isolated from all lines and RNA-seq was performed in replicate samples for each line (see Methods for additional details; data available at GEO Accession GSE94947). EdgeR bioinformatics analysis comparing WT cells to the three *HDAC2* null lines (5, 14, and 15) identified 95 genes differentially regulated at an EdgeR cut-off of 0.3 (Fig 3A and S6 Table). Comparing each individual HDAC2 null line to WT resulted in a very modest number of 315, 211, and 145 significantly differentially expressed genes, for lines 5, 14, and 15, respectively, at a p-value cutoff of 0.05 (Fig 3B). There was significant overlap of genes among each comparison of both two out of three and three out of three of the cell lines (Fig 3B). Of these genes, approximately equal numbers were upregulated and downregulated (Fig 3A and 3C). Since HDAC2 is primarily thought to repress genes, these results suggested that some downstream effects of *HDAC2* disruption are either indirect or due to the potential involvement of HDAC2 somewhat unexpectedly in direct activation of genes. Gene ontology analysis of genes affected in the combined analysis comparing WT and the three clonal lines demonstrated that genes upregulated by HDAC2 depletion are enriched in categories related to cell migration, cell signaling, and response to ionizing radiation (Fig 3D and S7 Table). Genes downregulated by HDAC2 depletion are enriched in categories related to collagen production and transmembrane transport (Fig 3E and S7 Table).

### HDAC2 is required for HDAC1 recruitment to direct targets of HDAC2 repression

**Fig 4:**
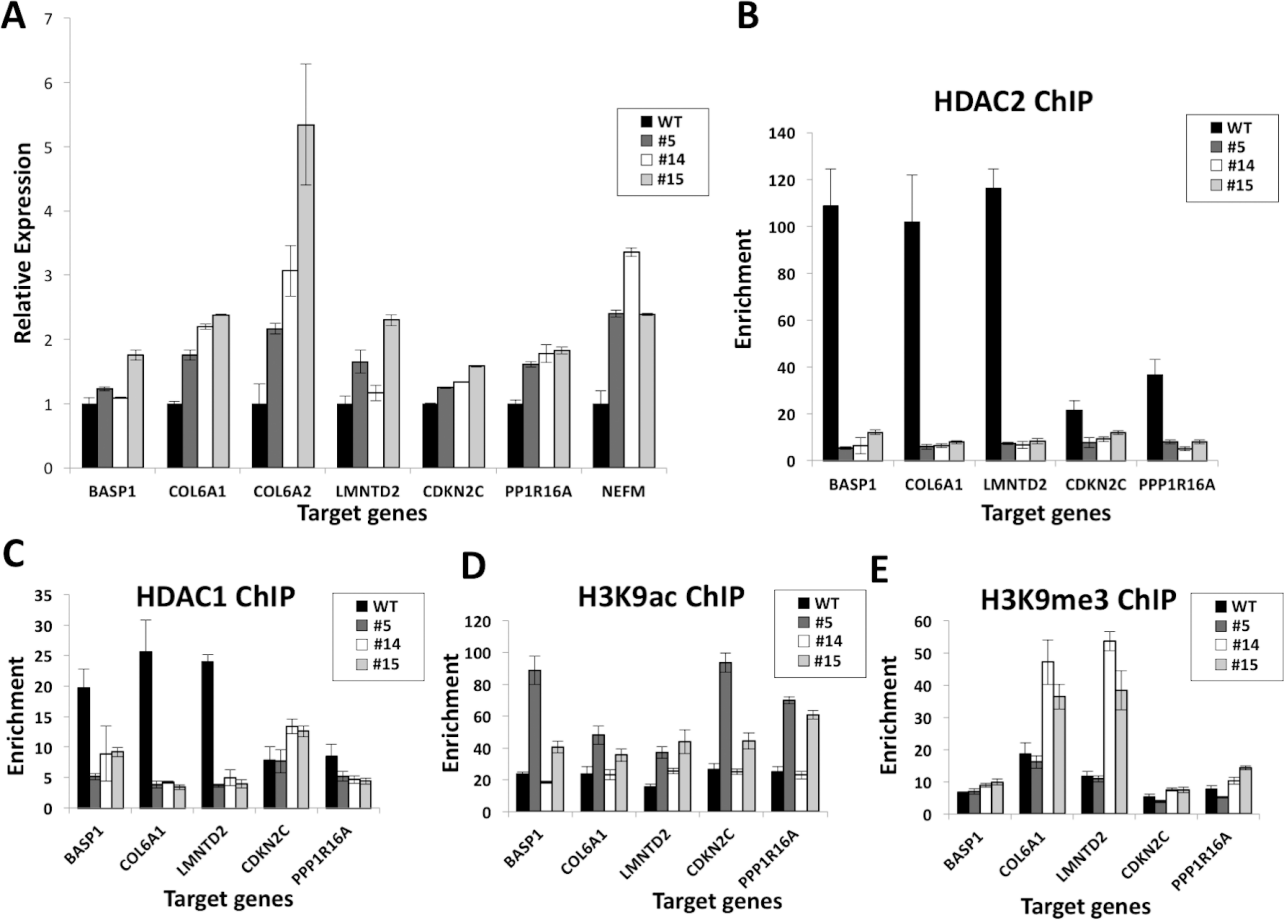
HDAC2 is required for HDAC1 recruitment to validated HDAC2-repressed target genes and in *HDAC2* nulls these targets display altered histone H3K9 modifications. **(A)** qPCR validation of candidate targets of HDAC2 repression as determined by RNA-seq. Gene expression of WT or the three clonal null lines (#5, #14 and #15) was internally normalized to GAPDH and fold enrichment represented relative to WT. All three null lines exhibited significantly elevated target gene expression for *COL6A2*, *LMNTD2*, *NEFM*, and *PPP1R16A* (p<0.05 in each case), and for *CDKN2C*and *COL6A1* (p<0.001 for both), but *BASP1* expression was significantly changed (p<0.05) only in clone #15. Error bars are S.E.M. **(B-F)** ChIP-qPCR was conducted on crosslinked chromatin of WT and clones #5, #14, and #15 with antibodies to HDAC2 **(B)**, HDAC1 **(C)**, H3K9ac **(D)**, H3K9me3 **(E)** or IgG control and qPCR analysis was conducted to evaluate the binding of HDAC2 or HDAC1 or enrichment of the specified histone marks at the promoter regions of the genes indicated. Enrichment was calculated by normalization to input and IgG control samples and plotted as mean of n = 3 with error bars representative of S.E.M.

As HDAC2 functions primarily in gene repression through its association with repressive complexes including the SIN3 complex [3,37], we chose high-confidence genes predicted to be upregulated by *HDAC2* disruption from the RNA-seq data (S6 Table), and conducted RT-qPCR validation analysis. All three *HDAC2* null lines displayed significant increases in (derepression of) the genes *COL6A1*, *COL6A2*, *LMNTD2*, *CDKN2C*, *PPP1R16A*, and *NEFM* (Fig 4A), consistent with previous reports that identified *COL6A2* and *CDKN2C* as genes that are upregulated by *HDAC2* knockdown in human cells [38,39]. *BASP1* was also upregulated in two clonal lines although it was identified as a predicted downregulated gene through RNA-seq. In order to test whether these are direct HDAC2 targets, ChIP assays were performed. We observed loss of HDAC2 binding in all three clonal lines compared to WT cells at most of the targets. *BASP1*, *CDKN2C*, *COL6A1*, *LMNTD2* and *PPP1R16A* (Fig 4B) were validated as direct targets of HDAC2-dependent repression. Because HDAC2 dimerizes with HDAC1 within many repressive complexes, we also examined whether loss of HDAC2 target binding affected HDAC1 binding at these genes as well. We found that at four out of five genes tested, loss of HDAC2 also resulted in substantial loss of HDAC1 binding in all three null lines (Fig 4C). These findings suggest that the recruitment of HDAC1 at repressed HDAC2 targets is dependent on the presence of HDAC2, probably via dimerization. An increase in HDAC1 enrichment was observed at *CDKN2C* in the HDAC2-null cells suggesting that it may be regulated through a different mechanism than the other HDAC2-null derepressed genes tested.

### Repressed HDAC2-target genes display increased H3K9ac as well as variably modified H4 acetylation and H3K9 methylation in nulls

We also tested whether derepression of genes due to loss of HDAC2 (most often leading to both reduced HDAC1 and HDAC2 target binding) also correlated with increased histone acetylation in their promoter regions. ChIP assays were conducted for pan-H4-acetylation (H4ac) and H3K9 acetylation (H3K9ac). We observed elevated H3K9ac with the loss of HDAC2 at all 5 derepressed genes tested (Fig 4D). We also conducted ChIP assays for H3K9me3, which is associated with gene repression. H3K9me3 was enriched in *HDAC2* null cells specifically at *COL6A1* and *LMNTD2* (Fig 4E). Additionally, we observed enrichment of H4ac over WT cells at two out of five derepressed genes tested (*BASP1* and *CDKN2C*) (S4A Fig). Overall, these ChIP data suggest that loss of *HDAC2* leads to alterations in histone mark levels at targets, including most prominently increased H3K9ac at HDAC2-repressed targets.

### A novel class of genes expressed in a partially HDAC2-dependent manner

**Fig 5:**
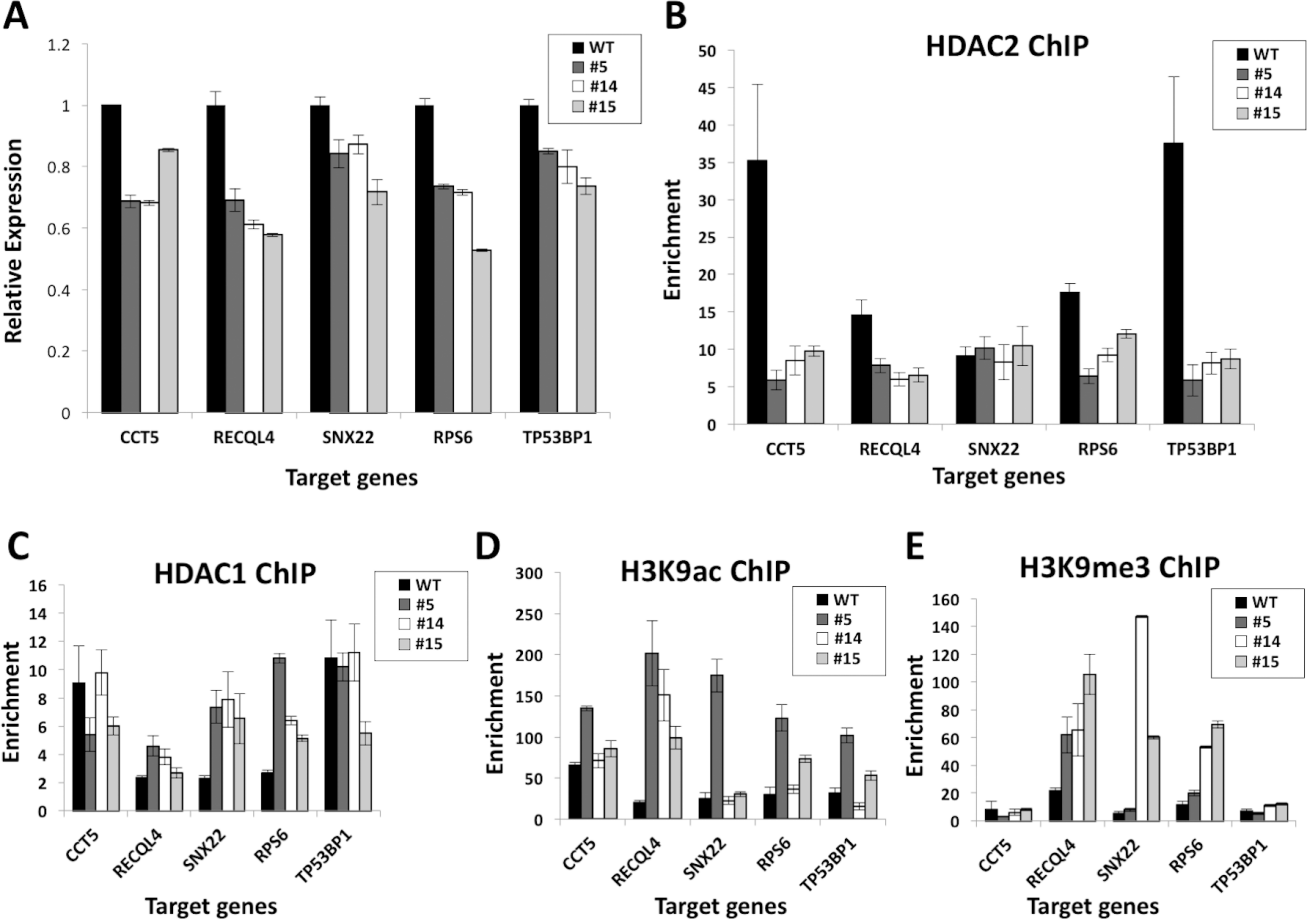
HDAC2-activated gene targets. **(A)** qPCR validation of candidate targets of HDAC2 activation as determined by RNA-seq. WT cells or *HDAC2* null clonal lines #5, #14 and #15 cDNA were analyzed by qPCR analysis in triplicates for transcript levels of the indicated candidate target genes. Gene expression was internally normalized to GAPDH and represented relative to WT. All three clonal lines exhibited decreased target gene expression with p<0.05 for *RECQL4*, *SNX22*, *TP53BP1*, and p<0.001 for *CCT5* and *RPS6*. **(B-E)** ChIP-qPCR was conducted on crosslinked chromatin of WT and clones #5, #14, and #15 with antibodies to HDAC2 **(B)**, HDAC1 **(C)**, H3K9ac **(D)**, H3K9me3 **(E)** or IgG control and SYBR green qPCR analysis was conducted to evaluate the binding of HDAC2 or HDAC1 or enrichment of the specified histone marks at the promoter regions of the genes indicated. Enrichment was calculated by normalization to input and IgG control samples and samples are plotted as mean of n = 3 with error bars representative of S.E.M.

We used qPCR to measure mRNA levels of specific genes predicted to be downregulated by loss of *HDAC2* by RNA-seq (S6 Table), observing a modest, but significant and consistent decrease in the expression of *CCT5*, *SNX22*, *RPS6*, and *TP53BP1* (Fig 5A), validating our RNA-seq findings. We also found *RECQL4*, although apparently upregulated approximately 1.5-fold by RNA-seq, to be consistently downregulated in *HDAC2* null cells by qPCR. To determine whether the reduced expression of these five genes was a direct or indirect effect of loss of *HDAC2*, we conducted ChIP assays that demonstrated loss of HDAC2 enrichment in *HDAC2*-null cells at each of these genes except *SNX22*, validating the remaining four as direct targets of HDAC2 that are decreased by its loss (Fig 5B). We also compared our observations of HDAC2 binding at the five repressed genes in *HDAC2* nulls, as well as binding at our validated derepressed gene targets in nulls to ENCODE HDAC2 ChIP-seq data from H1 ESC (GSM100372, GSM803345), K562 (GSM1003447, GSM803471) and MCF7 (GSM1010825) human cells [40]. Strikingly, all our defined direct HDAC2 targets were also HDAC2-bound according to ENCODE data in one or more other cell lines besides 293FTs, further supporting our findings overall and also specifically that the genes that are in some way activated in an HDAC2-dependent manner are bona fide direct targets of HDAC2 (S8 Table).

Notably, in contrast to the concomitant loss of HDAC1 binding that was observed for the derepressed genes (HDAC2 targets of repression) with disruption of *HDAC2*, the genes with reduced expression associated with disruption of *HDAC2* did not demonstrate a loss of HDAC1 binding (Fig 5C). Two of the five genes (*CCT5*, *TP53BP1*) tested had no consistent change in HDAC1 enrichment, indicating that these genes are not repressed in the nulls due to compensatory binding of HDAC1 in the absence of HDAC2. In contrast, an increase of HDAC1 enrichment was observed in the remaining three genes (*RECQL4*, *SNX22*, *RPS6*) tested. These data suggest that genes that are normally expressed in an at least partially HDAC2-dependent manner may not be bound by HDAC1-HDAC2 dimers and that at some of these targets HDAC1 may bind to partially compensate for loss of HDAC2.

To further validate that the effects on gene expression were specifically due to loss of HDAC2, and not due to the puromycin-based clonal selection process utilized to create the null cells, we compared the gene expression profile of all twelve validated genes from both target classes in WT parental cells and three clonal lines created through transfection of cells with “empty” Cas9 expressing plasmids without a guide RNA followed by puromycin selection. HDAC2-repressed targets displayed no significant increase in expression in the non-HDAC2 targeted clonal lines at any of the genes tested (S5A Fig) indicating that the elevated expression we observed in the *HDAC2* nulls (Fig 4A) was not due to puromycin clonal selection. In fact, these genes were mostly mildly downregulated in the clonal line controls. Additionally, three out of five genes of HDAC2-activated genes did not have significantly decreased gene expression in the clonal lines compared to WT (S5B Fig). However, *CCT5* and *RPS6* exhibited decreased expression suggesting that the clonal selection process may also play a role in the effects that we saw at these specific two genes.

### Increased H4ac, H3K9ac, and H3K9me3 at some HDAC2-activated target genes in nulls

To assess whether HDAC2-activated targets were associated with changes in histone modification marks, we examined pan-H4ac by ChIP assays at targets of HDAC2 activation. Despite the reduction in expression of HDAC2-activated genes in the *HDAC2* null cells, we unexpectedly found increased H3K9ac at four out of five genes examined (Fig 5D). Increased H3K9me3 was evident at three out of five genes tested (Fig 5E), which also correlated with the increase in HDAC1 DNA binding that was observed at these genes. Examination of pan-H4ac enrichment demonstrated some increased enrichment in clonal lines over WT cells at two of the genes tested (S4B Fig).

### Loss of HDAC2 does not alter global H3K9 or H4 modifications

Since some specific HDAC2 targeted genes demonstrated altered H4 and H3K9 acetylation patterns within their promoters, we tested whether CRISPR-mediated disruption of *HDAC2* could also alter global levels of these marks. Western blot analyses indicated that global H4ac, H3K9ac and H3K9me3 levels were not consistently altered in HDAC2-deficient clonal lines (S6A–S6F Fig).

### SIN3A binding is altered at some HDAC2-target genes

**Fig 6:**
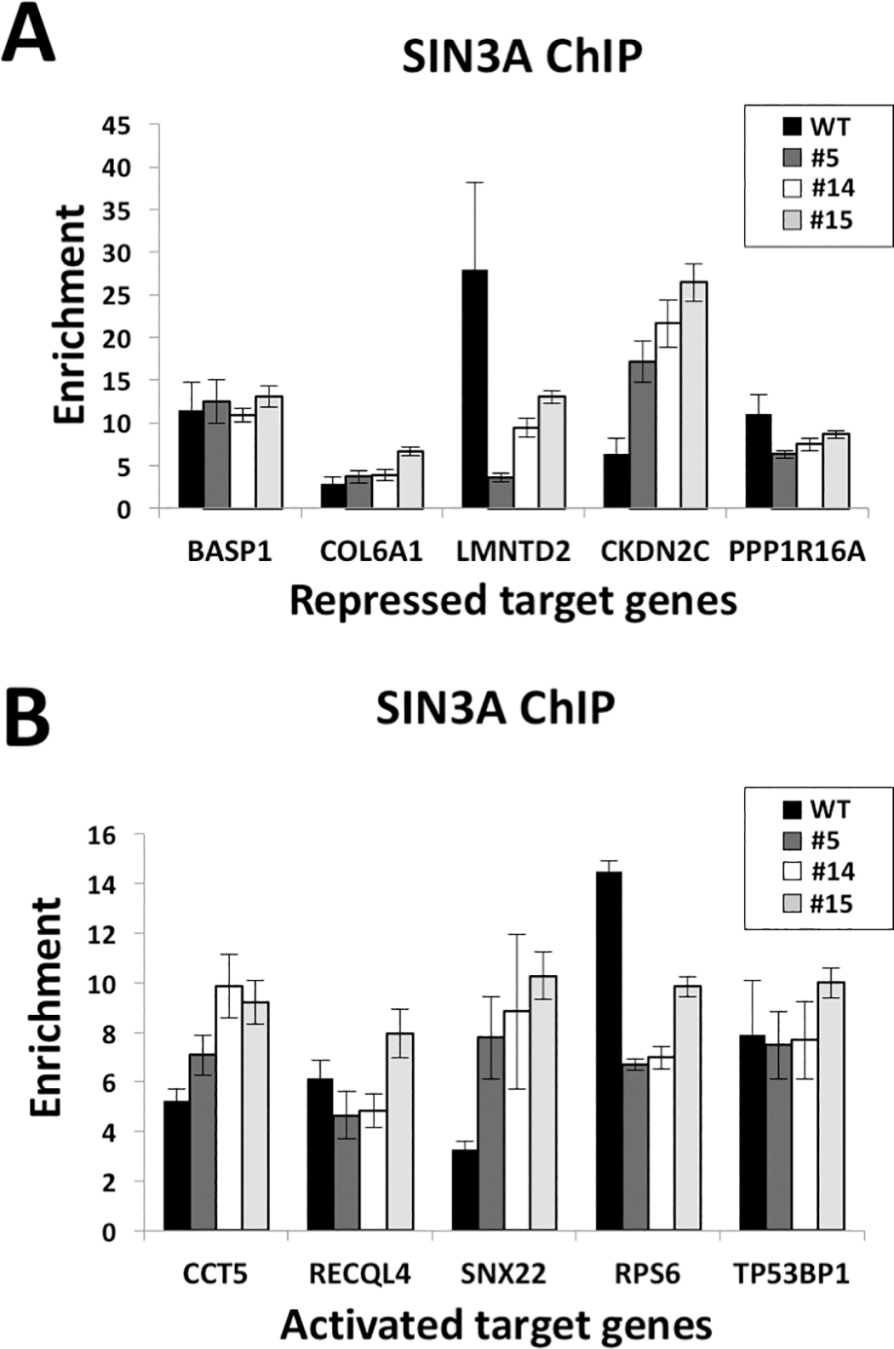
SIN3A binding at the two classes of HDAC2 target genes in *HDAC2* nulls. **(A-B)** ChIP-qPCR was conducted on crosslinked chromatin of WT, HDAC2 null clones #5, #14, and #15 with antibodies to SIN3A or IgG control and qPCR analysis was conducted to evaluate the binding of SIN3A at the promoter regions of HDAC2-repressed gene targets **(A)** or HDAC2-activated gene targets **(B).** Enrichment was calculated by normalization to input and IgG control samples and plotted as mean of n = 3 with error bars representative of S.E.M.

We assessed whether the loss of *HDAC2*, and in some cases the concomitant loss of HDAC1 binding at target genes, altered binding of the HDAC-associated co-repressor SIN3A to these genes. ChIP assays for SIN3A indicated that six out of ten genes tested (*LMNTD2*, *CDKN2C*, *PPP1R16A*, *CCT5*, *SNX22*, *RPS6*) exhibited altered SIN3A enrichment in *HDAC2*-null cells, but without a clear pattern of decreased versus increased binding in the nulls (Fig 6A and 6B). For instance, the HDAC2-repressed gene *LMNTD2* displayed a marked loss of SIN3A binding in HDAC2 nulls compared to WT cells, but for another repressed gene *CDNK2C* an increase in SIN3A was evident in nulls (Fig 6A). At genes repressed by loss of HDAC2, increased SIN3A enrichment was also evident in the case of *CCT5* and *SNX22*, while *RPS6* had decreased enrichment at SIN3A (Fig 6B). Overall, as predicted (since SIN3A is recruited to specific targets by transcription factors and not HDACs), loss of HDAC2 did not fully disrupt SIN3A binding at any target. However, the variable changes in SIN3A occupancy that we did observe suggest that loss of HDAC2 can impact SIN3A complex binding or stability in some instances.

## Discussion

Although it may be tempting to view HDACs and HATs in a simple, antagonistic manner with the former strictly mediating repression and the latter activation, the contributions of these enzymes to epigenomic control mechanisms are more complicated than a simple binary model would predict. For instance, here we identified a class of genes to which HDAC2 contributes some kind of activating function. HDAC2’s role in expression of specific actively transcribed genes, although modest, was very reproducible. Inhibition of the enzymatic activity of HDACs reportedly affects only 5–20% of total gene expression with approximately equal changes in the numbers of upregulated and downregulated genes [41]. This is consistent with both the relatively low proportion of total gene expression changes that we found with HDAC2 depletion in 293FT cells, and the notion of HDAC2 having a distinct function contributing to gene activity. However, mechanisms by which HDACs could actively contribute to gene transcription remain somewhat obscure. One notion is that it could be a consequence of the requirement for HDAC2 at specific types of targets for prevention of inappropriate re-initiation of transcription [12], which may take place in *HDAC2* nulls at certain target genes, could yield overall transcription below the normal magnitude.

**Fig 7:**
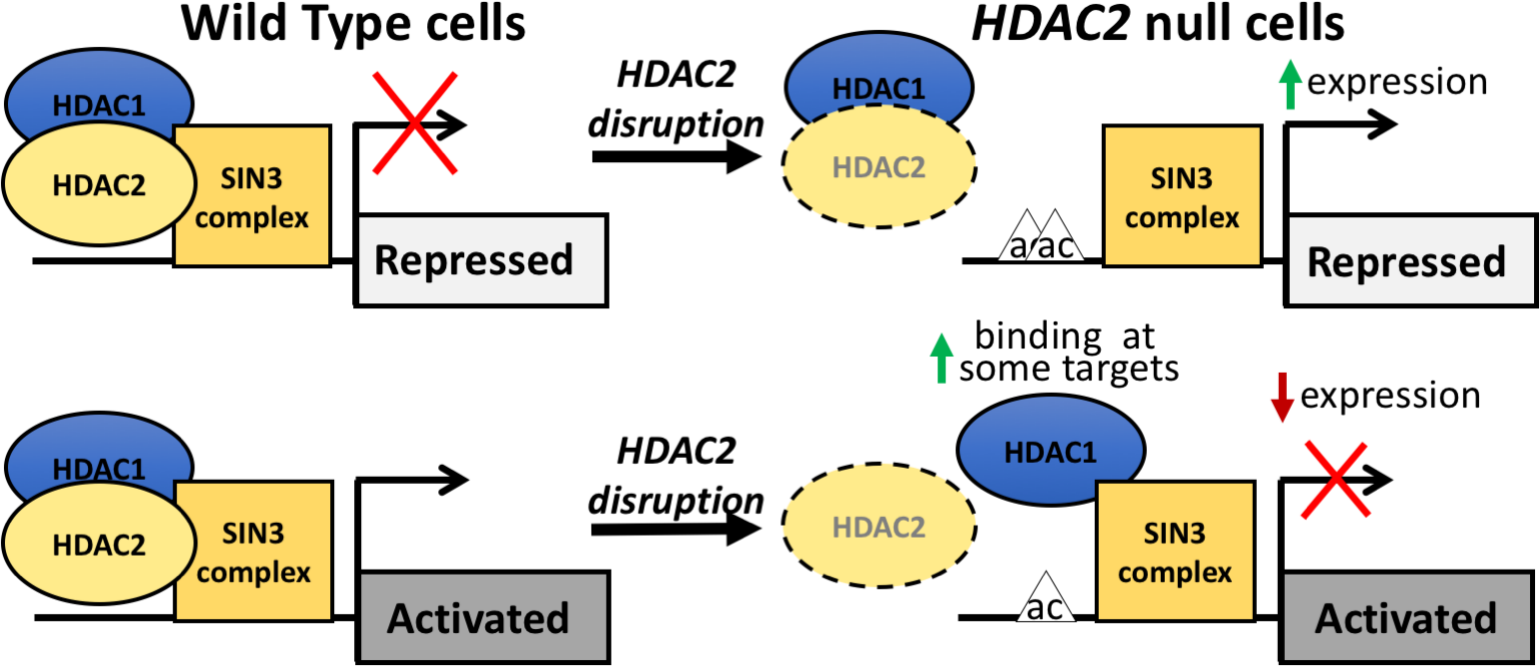
An overall model of the two classes of HDAC2-target genes. HDAC2-repressed gene targets require the presence of HDAC2 for both HDAC2 and HDAC1 binding at gene targets as evident by the loss of enrichment of both with disruption of *HDAC2*. In the second class, novel HDAC2-activated target genes, HDAC2 is not necessary for the recruitment of HDAC1 to target genes. In both classes of gene targets, SIN3 enrichment is variably altered but not completely disrupted. Varying degrees of increases in histone acetylation, mainly at H3K9, at gene targets is observed with loss of *HDAC2*.

Additional data from our study supports a model of two distinct classes of HDAC2 targets (Fig 7). We found that while HDAC2 and HDAC1 co-bind HDAC2 targets of repression putatively through dimerization in which HDAC1 is dependent on HDAC2, targets of HDAC2 activation did not exhibit this dependence. Therefore, we predict either that HDAC2 contributes to gene activation independently of the HDAC1-HDAC2 dimer that is required for repression or that independent HDAC1 binding robustly compensates for loss of dimer binding. In either case in this kind of model, the data suggest that HDAC2 could be present at these targets in a distinct protein complex, perhaps with different histone modifying enzymatic activity. Although we found evidence that SIN3A bound to some HDAC2 targets of activation, it is also possible that HDAC2 is present in multiple distinct protein complexes even at individual discrete target genes. There is precedent for SIN3 complex constituents promoting transcriptional activation. A recent study identified the SIN3A-HDAC2 complex as co-occupying transcriptionally active promoters with NANOG in mouse ESCs [42]. Additionally, several components of the HDAC1/2-containing NuRD complex, such as MTA1, have been found to promote gene activation [43,44] in specific cellular contexts. It will be of considerable interest in future studies to investigate whether additional co-repressor complexes such as NuRD and Co-REST play roles at the HDAC2 targets we identified and studied here.

Notably, upon HDAC2 disruption, increased HDAC1 binding was observed at some active targets suggesting that although the two potentially do not dimerize at these targets HDAC1 can nonetheless be compensatory. Previous studies have also reported a significant number of genes with more than two-fold downregulation of expression in *Hdac1* KO mouse ESCs that are associated with increased recruitment of HDAC2 at these genes [23]. Since we observed that subsets of genes that are downregulated upon HDAC2-disruption either have unchanged or increased HDAC1 enrichment compared to WT cells, what we term as “over-compensation” by HDAC1 could occur. In this scenario, increased HDAC1 binding or activity in the absence of HDAC2 above physiological levels may be the mechanism of decreased expression of these genes. In contrast, the genes that have no observable change in HDAC1 occupancy in *HDAC2* nulls may be more confidently viewed as bona fide HDAC2 targets of activation that exhibit repression due its disruption. While we found evidence that by itself the clonal selection process using puromycin may have contributed to the decreased gene expression of two out of five genes in the HDAC2-activated class of genes in *HDAC2* nulls, we still found loss of HDAC2 enrichment at both genes and a concomitant increase in HDAC1 enrichment at *RPS6*, indicating that they are indeed direct targets of HDAC2. The further validation of our specific active gene targets as HDAC2-bound in various human cell types by ENCODE ChIP-seq data also supports our hypothesis of an active class of target genes and suggests that these targets are important more broadly across a variety of human cells beyond just 293FTs. However, to our knowledge, there are no reports of validation and analysis of specific gene targets that HDAC2 directly activates with which to compare our findings.

Our observation that HDAC2 binding is necessary for the recruitment of HDAC1 to repressed gene targets raises the question of why targeted disruption of *Hdac2* does not produce a more pronounced murine knockout phenotype? One possibility is that targeted disruption of *Hdac2* in an *in vivo* developmental context leads to more robust *Hdac1* compensation or reveals differences in Hdac1-Hdac2 redundancy compared to what we observed in the human cellular context in 293FT cells. *Hdac1* disruption also appears to produce more pronounced phenotypes in a cell context-dependent manner than disruption of *Hdac2* [7,11,16], suggesting some distinct developmental functions for the two proteins and a lack of complete compensatory potential. Consistent with this notion, we found that increased HDAC1 binding following the loss of HDAC2 at the *CDKN2C* gene was still not sufficient to prevent its derepression, indicating that an increase in HDAC1 enrichment cannot always functionally compensate for HDAC2. Studies in *Hdac1* KO ESCs have also demonstrated that Hdac2 deacetylase activity can be increased in the absence of Hdac1, demonstrating a compensatory function for Hdac2 even in the absence of its dimerization partner [23]. That study found that at certain repressed gene targets of Hdac1, Hdac2 can compensate for the loss of Hdac1 leading to increased H3 and H4 acetylation at these genes. Additionally, increased H3K9 acetylation has been associated with selective kinetic inhibition of Hdac2 in mouse neuronal cultures [45]. Our findings of H3K9 acetylation enrichment at gene targets in HDAC2-null cells correlate with these studies. Both in the human and mouse context, the question arises of whether HDAC2 or HDAC1 homodimers can replace HDAC1-HDAC2 heterodimers, and if so, what the distinct function of the heterodimer might be over the homodimer. It is possible that the temporal recruitment of HDAC1 versus HDAC2 by selective transcription factors or specific co-repressor complexes determines the ultimate effect on expression levels of target genes. This is further supported by studies reporting HDAC1/2 heterodimers and independent HDAC2 protein to be present at different levels depending on the cellular context [10,11]. Additionally, HDAC2 was found to be bound to promoters of specific neuronal plasticity-associated genes independent of HDAC1, further supporting an individual role for HDAC2 in certain physiological contexts such as in cognitive function [46].

Our study also potentially invokes other histone modifying complexes besides those regulating acetylation. Our finding that increased H3K9 methylation correlates with increased enrichment of HDAC1 at repressed genes in *HDAC2*-disrupted cells, but not at derepressed genes, suggests functional ties between HDAC2 and enzymes regulating histone methylation, and it further supports the existence of some independent functions for HDAC1 and HDAC2 at this set of genes. Zupkovitz, *et al*. also demonstrated a link between increased HDAC1 expression and elevated H3K9me3 in *Hdac1*-KO mouse embryonic stem cells [23]. It is notable that they observed a decrease in H3K9me3 with *Hdac1* KO at repressed gene targets, in contrast to our study where we find an increase in H3K9me3 at some repressed targets in the absence of HDAC2. The H3K9me3 change we observed could be due to increased histone methylation and/or decreased histone demethylase activity that is affected by levels of HDAC2 and/or HDAC1. Accordingly, a physical association between HDAC1 and the histone methyltransferase, Suv39h1 [47], and interaction of both HDAC1 and HDAC2 with the SETDB1 histone methyltransferase that specifically methylates H3 at Lysine 9 [48] support this notion. ChIP-seq studies for other specific histone marks in the HDAC2 null 293FT cells should provide further insights into histone modification changes and regulatory mechanisms.

## Conclusions

Overall, we have established a requirement of HDAC2 in proper regulation of gene repression, demonstrated its relatively more novel role in active gene expression, and identified two distinct classes of HDAC2-bound and regulated targets. Our study provides new insights into functions of HDAC2 in human cells that are both conserved within the Class I HDAC family and unique to HDAC2, which may aid future studies in distinguishing the precise roles of HDAC2 in contributing to certain types of cancer and in normal development. Together, our work here, other recent studies on a wider range of Class I HDACs including in cancer [49], and future studies will provide broader insights into class I HDAC functions overall.

### Availability of data

RNA-seq data has been deposited in GEO (Accession number GSE94947).

## Supporting information

S1 Fig PCA of WT and clonal lines. The DESeq2 R package was used to perform and plot PCA.
(TIF)

S2 Fig *HDAC2* disruption reduces HDAC3, but not HDAC1: Individual clone data. **(A)** Quantitation of individual clones shown in Fig 1B. HDAC2 protein levels normalized to B-actin were quantified through the LiCOR imaging software and plotted relative to WT. **(B)** Nuclear lysates of a panel of 9 additional HDAC2-targeted clonal lines were analyzed for HDAC2 protein levels through Western blotting. **(C)** Quantitation of panel B. HDAC2 protein levels were plotted relative to WT. **(D)** Nuclear lysates of a panel of 13 HDAC2-targeted clonal lines, including the three characterized lines #5, #14, and #15, were analyzed for HDAC1 protein levels through Western blotting. **(E)** Quantitation of panel D. HDAC1 protein levels were plotted relative to WT. Mean quantitation is represented in Fig 1D. **(F)** Nuclear lysates of a panel of 13 HDAC2-targeted clonal lines, including the three characterized lines #5, #14, and #15, were analyzed for HDAC3 protein levels through Western blotting. **(G)** Quantitation of panel F. HDAC3 protein levels were plotted relative to WT. Mean quantitation is represented in Fig 1E. All protein levels were normalized to B-actin.
(TIF)

S3 Fig Predicted off-target regions do not have Indels. Genomic DNA was isolated from WT cells or *HDAC2* null clones #5, #14, and #15 and PCR-amplified for the top four predicted off-target regions based on sequence complementarity to the gRNA sequence, as predicted by the website tool. The predicted off-target regions in intronic regions of *BMP15* **(A)**, *PARP2* **(B)**, *MYL2* **(C)**, or *IKBKB* **(D)** were sequenced. All alignments were done to the hg19 reference genome. Red boxes indicate the predicted off-target sequence within the alignment.
(TIF)

S4 Fig HDAC2 null lines exhibit increased H4ac enrichment at a few target genes. ChIP-qPCR was conducted on crosslinked chromatin of WT and clones #5, #14, and #15 with antibodies to pan-H4ac or IgG control and SYBR green qPCR analysis was conducted for HDAC2-repressed **(A)** or HDAC2-activated **(B)** gene targets to evaluate the binding of HDAC2 or HDAC1 or enrichment of the specified histone marks at the promoter regions of the genes indicated. Enrichment was calculated by normalization to input and IgG controls and samples are plotted as mean of n = 3 with error bars representative of S.E.M.
(TIF)

S5 Fig Expression patterns of HDAC2 target genes are not consistently altered in the same ways simply by clonal selection and puro exposure absent HDAC2 disruption as compared to RNA-Seq defined expression changes in HDAC2 knockouts. RNA isolated from 293FT cells transfected with empty pCas9-puro and clonally selected with puromycin was subjected to qPCR analysis for expression of genes found to be upregulated **(A)** with HDAC2-disruption (see Fig 4A) or downregulated **(B)** with HDAC2-disruption (see Fig 5A). Gene expression of WT or the three clonal lines were internally normalized to GAPDH and the average fold enrichment of three clonal lines are represented relative to WT. Error bars represent S.E.M. and * denotes p<0.05. KO = knockout.
(TIF)

S6 Fig Loss of HDAC2 does not alter global H3K9 or H4 modifications. **(A-C)** Histone acid extracts of WT or *HDAC2* null clones #5, #14, and #15 were analyzed for total H4ac **(A)**, H3K9ac **(B)**, or H3K9me3 **(C)** levels through Western blotting. Image is representative of three independent Western blots. **(D-F)** Quantitation of the triplicate Western blot results: H4ac **(D)**, H3K9ac **(E)**, and H3K9me3 **(F)**. In each case, protein levels were normalized to B-actin with LiCOR imaging software and represented relative to WT with error bars S.E.M (n = 3).
(TIF)

S1 Table Off-target region PCR primers. (DOCX)

S2 Table Tested off-target sites as predicted by crispr.mit.edu. (DOCX)

S3 Table Quality of RNA-Seq reads as determined through RSeQC. Duplication rates (Dup) were determined both by Position (Pos) and Sequence (Seq).
(DOCX)

S4 Table qPCR assay primers. (DOCX)

S5 Table ChIP-qPCR assay primers. (DOCX)

S6 Table Differentially expressed genes in HDAC CRISPR lines. (XLSX)

S7 Table GO analysis of HDAC2 upregulated and downregulated genes. (XLS)

S8 Table ENCODE HDAC2 ChIP-seq peaks in Tier 1 cell lines present at validated HDAC2 targets. (DOCX)
