# Supplementary figures and images for: CRISPR-mediated HDAC2 disruption identifies two distinct classes of target genes in human cells
Source: PLoS One. 2017 Oct 5;12(10):e0185627. doi: 10.1371/journal.pone.0185627 (PMC5628847; doi:10.1371/journal.pone.0185627)

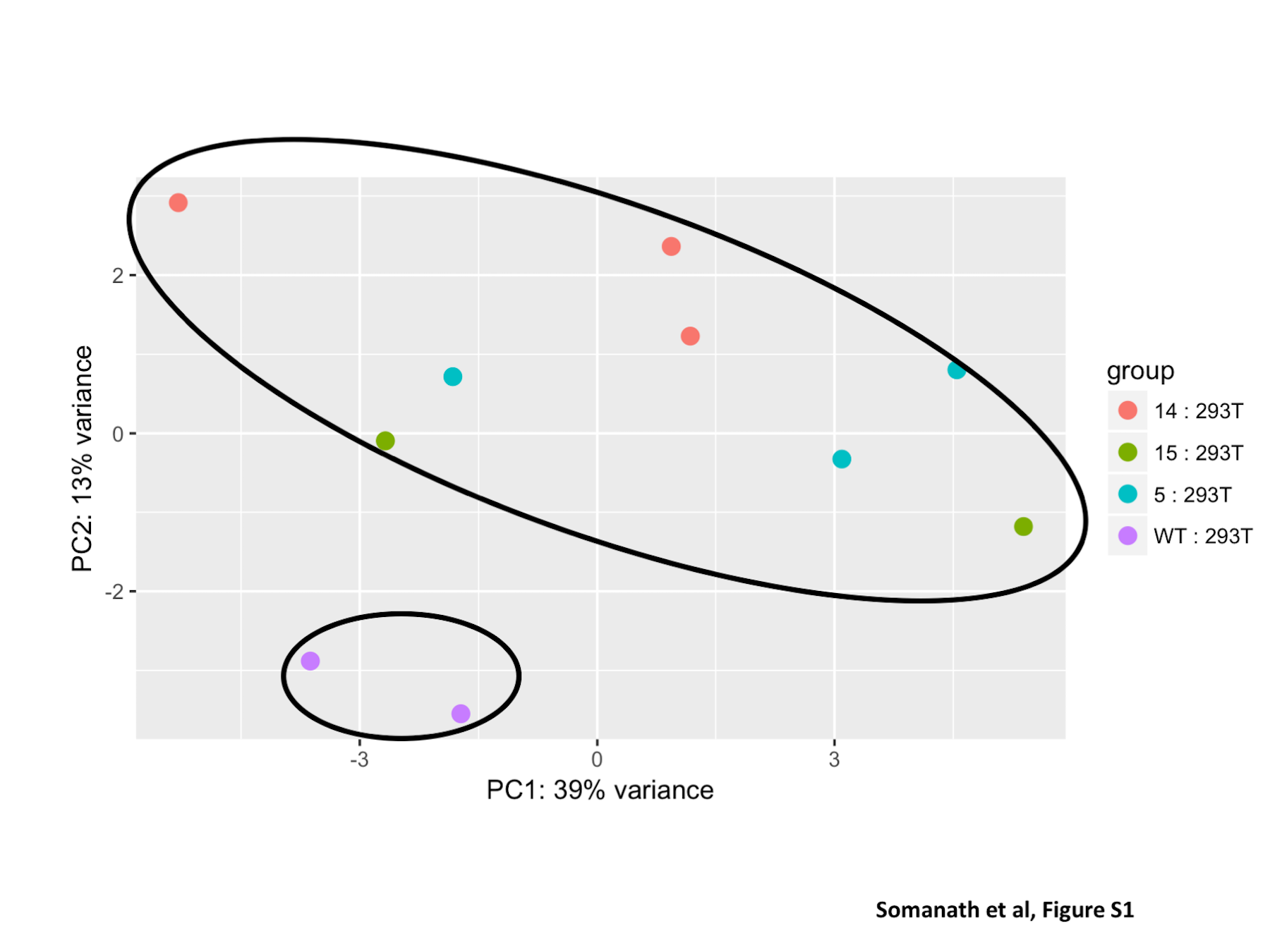

Supplement: S1 Fig — The DESeq2 R package was used to perform and plot PCA. (TIF) [file pone.0185627.s001.tif]

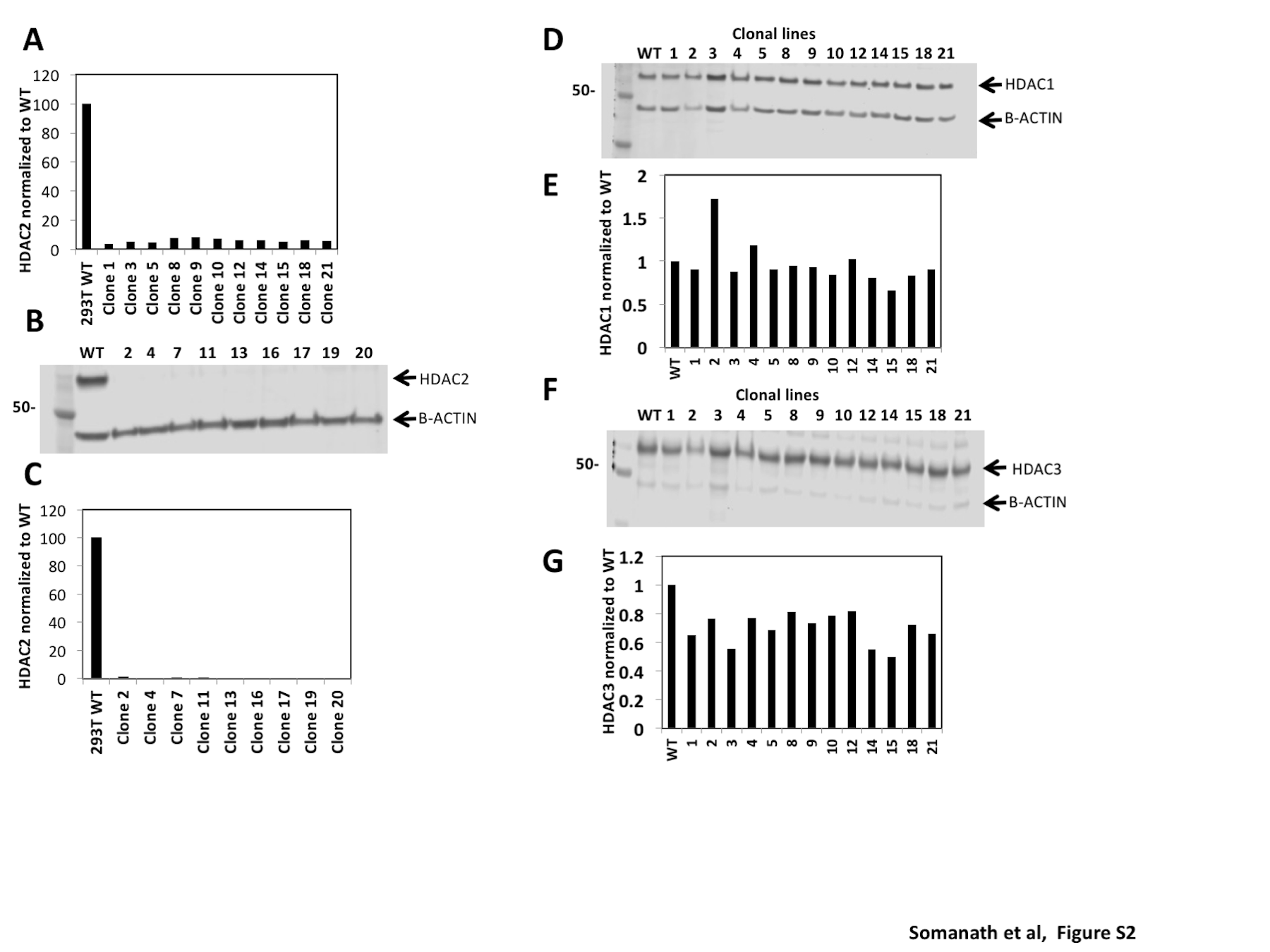

Supplement: S2 Fig — (A) Quantitation of individual clones shown in Fig 1B. HDAC2 protein levels normalized to B-actin were quantified through the LiCOR imaging software and plotted relative to WT. (B) Nuclear lysates of a panel of 9 additional HDAC2-targeted clonal lines were analyzed for HDAC2 protein levels through Western blotting. (C) Quantitation of panel B. HDAC2 protein levels were plotted relative to WT. (D) Nuclear lysates of a panel of 13 HDAC2-targeted clonal lines, including the three characterized lines #5, #14, and #15, were analyzed for HDAC1 protein levels through Western blotting. (E) Quantitation of panel D. HDAC1 protein levels were plotted relative to WT. Mean quantitation is represented in Fig 1D. (F) Nuclear lysates of a panel of 13 HDAC2-targeted clonal lines, including the three characterized lines #5, #14, and #15, were analyzed for HDAC3 protein levels through Western blotting. (G) Quantitation of panel F. HDAC3 protein levels were plotted relative to WT. Mean quantitation is represented in Fig 1E. All protein levels were normalized to B-actin. (TIF) [file pone.0185627.s002.tif]

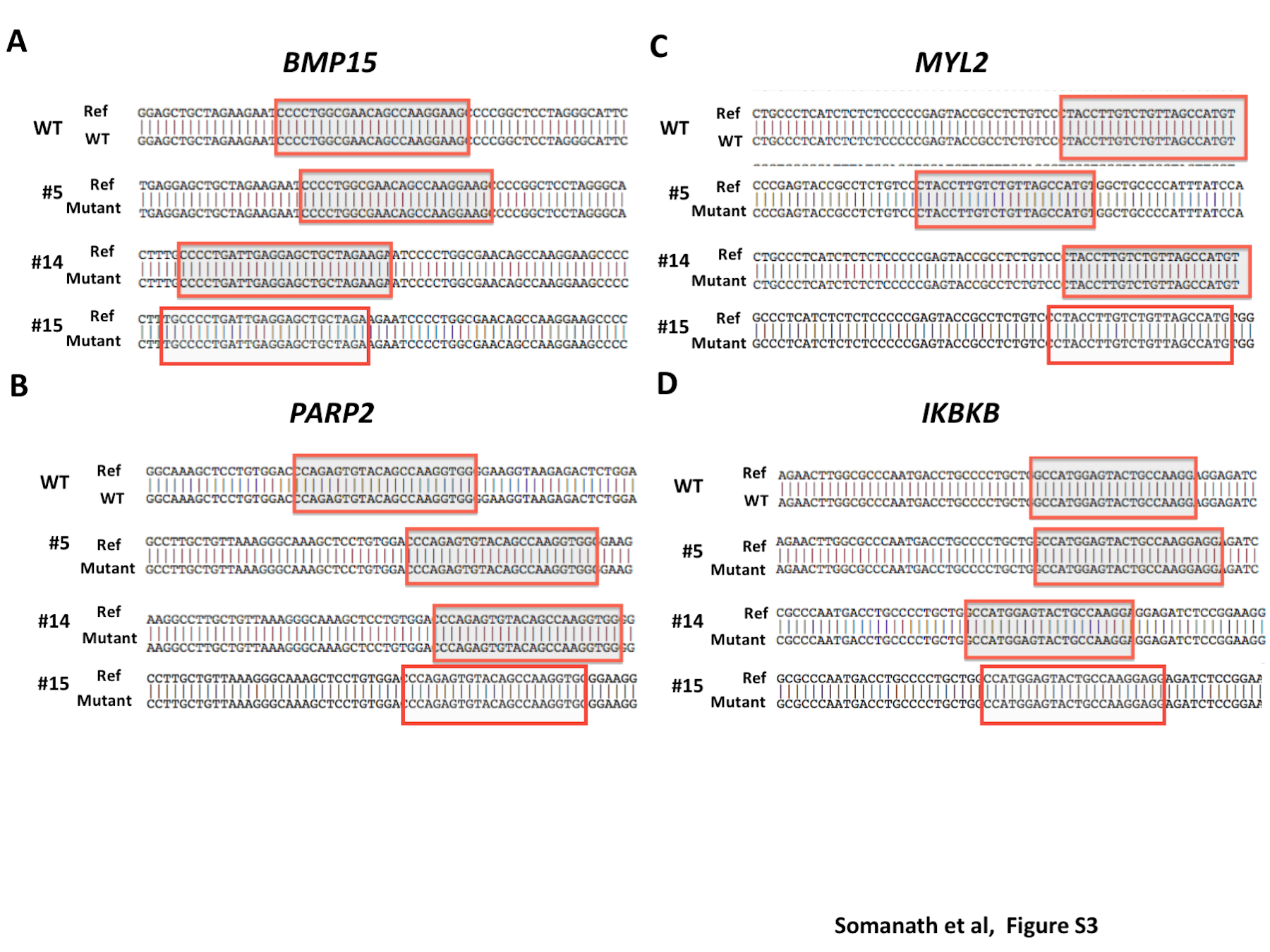

Supplement: S3 Fig — Genomic DNA was isolated from WT cells or HDAC2 null clones #5, #14, and #15 and PCR-amplified for the top four predicted off-target regions based on sequence complementarity to the gRNA sequence, as predicted by the website tool. The predicted off-target regions in intronic regions of BMP15 (A), PARP2 (B), MYL2 (C), or IKBKB (D) were sequenced. All alignments were done to the hg19 reference genome. Red boxes indicate the predicted off-target sequence within the alignment. (TIF) [file pone.0185627.s003.tif]

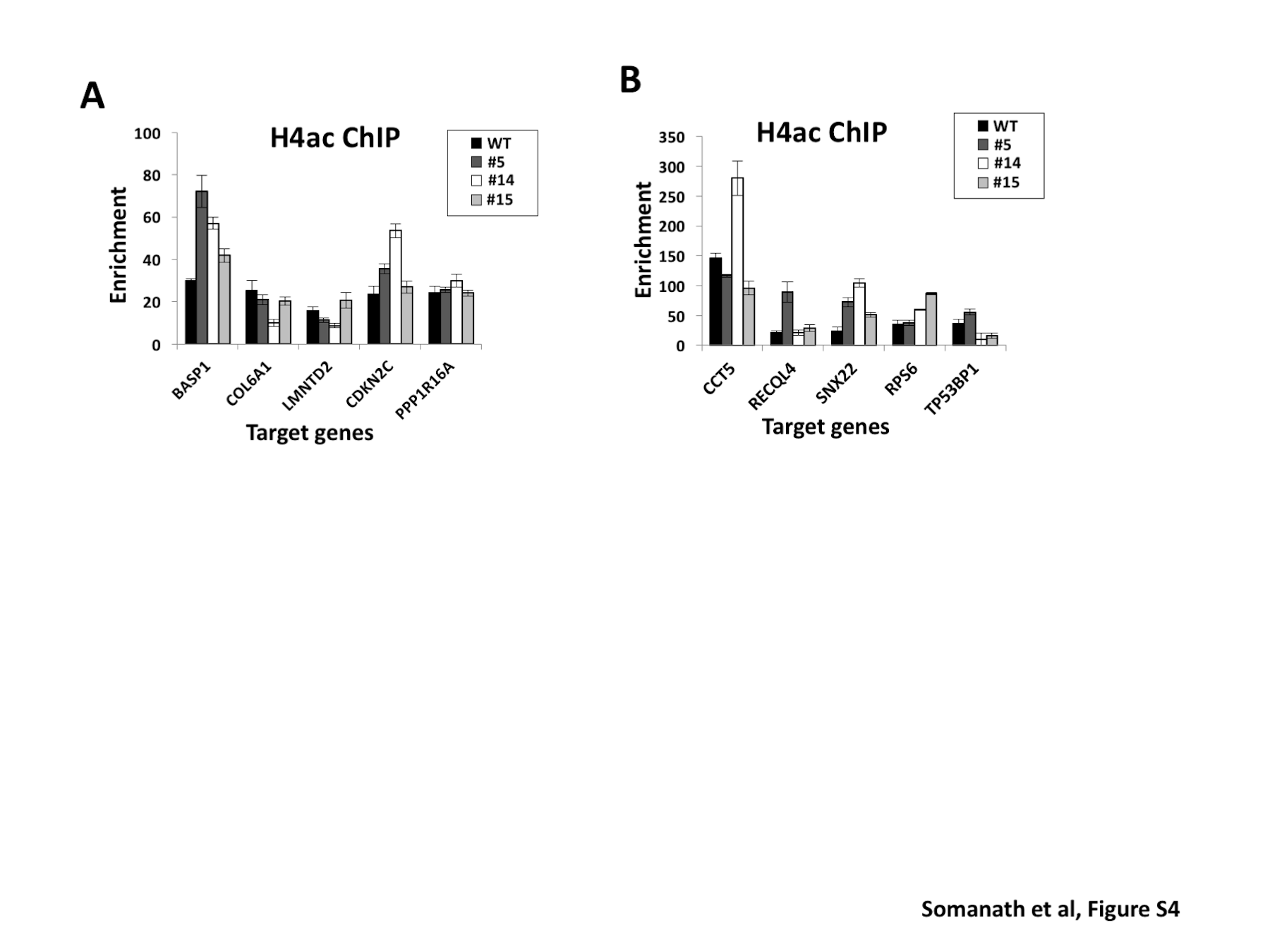

Supplement: S4 Fig — ChIP-qPCR was conducted on crosslinked chromatin of WT and clones #5, #14, and #15 with antibodies to pan-H4ac or IgG control and SYBR green qPCR analysis was conducted for HDAC2-repressed (A) or HDAC2-activated (B) gene targets to evaluate the binding of HDAC2 or HDAC1 or enrichment of the specified histone marks at the promoter regions of the genes indicated. Enrichment was calculated by normalization to input and IgG controls and samples are plotted as mean of n = 3 with error bars representative of S.E.M. (TIF) [file pone.0185627.s004.tif]

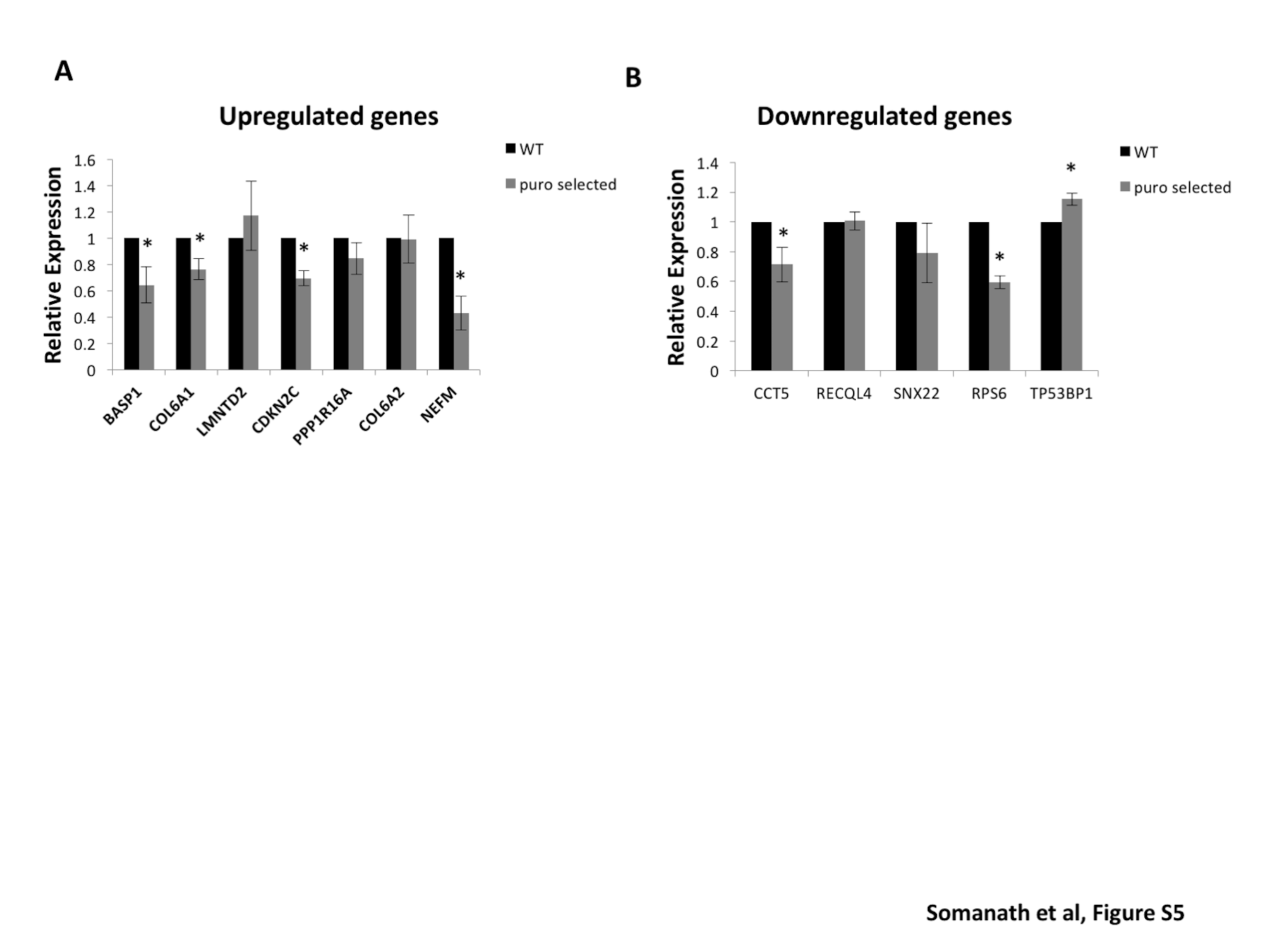

Supplement: S5 Fig — RNA isolated from 293FT cells transfected with empty pCas9-puro and clonally selected with puromycin was subjected to qPCR analysis for expression of genes found to be upregulated (A) with HDAC2-disruption (see Fig 4A) or downregulated (B) with HDAC2-disruption (see Fig 5A). Gene expression of WT or the three clonal lines were internally normalized to GAPDH and the average fold enrichment of three clonal lines are represented relative to WT. Error bars represent S.E.M. and * denotes p<0.05. KO = knockout. (TIF) [file pone.0185627.s005.tif]

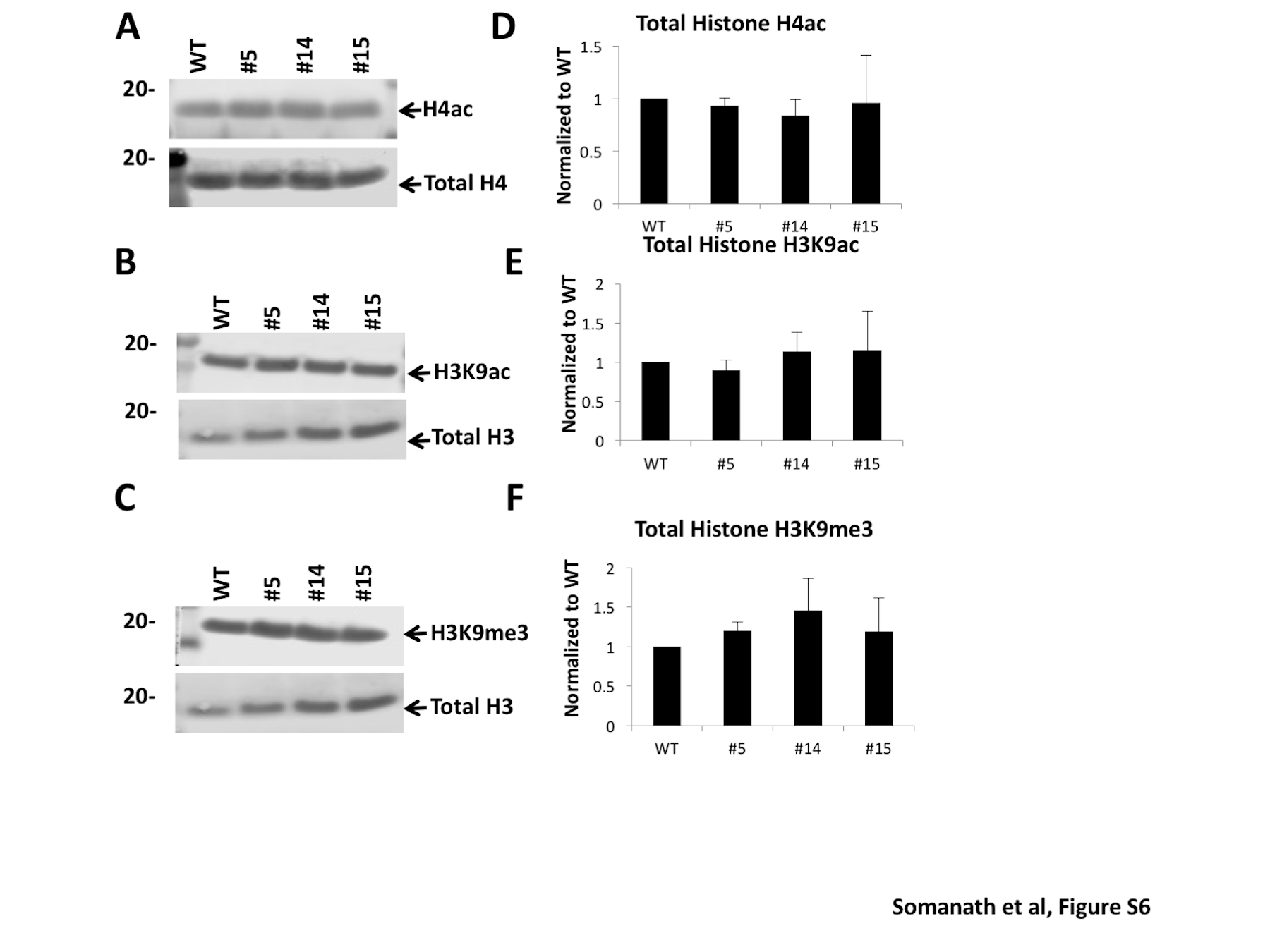

Supplement: S6 Fig — (A-C) Histone acid extracts of WT or HDAC2 null clones #5, #14, and #15 were analyzed for total H4ac (A), H3K9ac (B), or H3K9me3 (C) levels through Western blotting. Image is representative of three independent Western blots. (D-F) Quantitation of the triplicate Western blot results: H4ac (D), H3K9ac (E), and H3K9me3 (F). In each case, protein levels were normalized to B-actin with LiCOR imaging software and represented relative to WT with error bars S.E.M (n = 3). (TIF) [file pone.0185627.s006.tif]
