# Supplementary material for: CRISPR-mediated HDAC2 disruption identifies two distinct classes of target genes in human cells
Source: PLoS One. 2017 Oct 5;12(10):e0185627. doi: 10.1371/journal.pone.0185627 (PMC5628847; doi:10.1371/journal.pone.0185627)
Supplement: S1 Table — (DOCX) [file pone.0185627.s007.docx]

**Somanath et al, Supplementary Information**

**S1 Table. Off-target region PCR primers**

| **Region** | **Forward Primer** | **Reverse Primer** |
| --- | --- | --- |
| *BMP15* | TTGTGTTGGGGCCTGTTGT | GGGGCATAACAACTCACCTCT |
| *PARP2* | GCGGGCAAAGTTAATCCTGAC | AGCCCTACCACTCCTATCTCT |
| *MYL2* | TTGCTGGCTCATTGCAGGTT | TCCAACTGTAGGATGTGCGG |
| *IKBKB* | TCAAAGCCCTGGGTTATCCT | ACAGAAGTGAAAGGTCTCCCCA |
