# Supplementary material for: CRISPR-mediated HDAC2 disruption identifies two distinct classes of target genes in human cells
Source: PLoS One. 2017 Oct 5;12(10):e0185627. doi: 10.1371/journal.pone.0185627 (PMC5628847; doi:10.1371/journal.pone.0185627)
Supplement: S2 Table — (DOCX) [file pone.0185627.s008.docx]

**Somanath et al, Supplementary Information**

**S2 Table. Tested off-target sites as predicted by crispr.mit.edu.**

| **Gene** | **Sequence** | **Score** | **Mismatches** |
| --- | --- | --- | --- |
| NM_005448 | CCCCTGGCGAACAGCCAAGGAAG | 0.7 | 3 (4:10:15) |
| NM_001042618 | CCCAGAGTGTACAGCCAAGGTGG | 0.2 | 4 (5:6:8:15) |
| NM_000432 | CACATGGCTAACAGACAAGGTAG | 0.2 | 4 (2:9:10:15) |
| NR_04009 | GCCATGGAGTACTGCCAAGGAGG | 0.2 | 4 (4:7:9:15) |
