# Supplementary material for: CRISPR-mediated HDAC2 disruption identifies two distinct classes of target genes in human cells
Source: PLoS One. 2017 Oct 5;12(10):e0185627. doi: 10.1371/journal.pone.0185627 (PMC5628847; doi:10.1371/journal.pone.0185627)
Supplement: S3 Table — Duplication rates (Dup) were determined both by Position (Pos) and Sequence (Seq). (DOCX) [file pone.0185627.s009.docx]

**Somanath et al, Supplementary Information**

**S3 Table. Quality of RNA-Seq reads as determined through RSeQC.**

| **Sample** | **Total Reads** | **Mapped Reads** | **CDS Exons Tags** | **Intron Tags** | **5’ UTR Exon Tags** | **3’ UTR Exon Tags** | **Dup Rate (Pos)** | **Dup Rate**  **(Seq)** |
| --- | --- | --- | --- | --- | --- | --- | --- | --- |
| WT-1 | 35280330 | 31347513 | 20066464 | 3126112 | 1063795 | 7394058 | 81.1% | 74.6% |
| WT-2 | 23907971 | 21729495 | 14415695 | 1934254 | 733092 | 5137904 | 76.9% | 70.0% |
| WT-3 | 30489156 | 26568811 | 15304957 | 3173613 | 956602 | 5819301 | 86.1% | 82.2% |
| 5-1 | 32737433 | 29624796 | 19532664 | 2632924 | 1031143 | 6856659 | 81.3% | 74.8% |
| 5-2 | 32959656 | 29593340 | 18768782 | 3151631 | 1035205 | 6976302 | 79.4% | 72.9% |
| 5-3 | 36460423 | 31923452 | 19660509 | 3754277 | 1115929 | 7385758 | 79.9% | 73.4% |
| 14-1 | 30510676 | 27250054 | 17939547 | 2393864 | 995658 | 6298821 | 80.6% | 73.7% |
| 14-2 | 33099768 | 29038905 | 18551237 | 3174646 | 1053942 | 6488578 | 79.8% | 73.2% |
| 14-3 | 35451569 | 32728798 | 21178267 | 3247996 | 1091129 | 7695880 | 80.4% | 73.5% |
| 15-1 | 32266344 | 29428508 | 18981831 | 2631124 | 1061185 | 6949898 | 80.1% | 73.2% |
| 15-2 | 40809379 | 37787290 | 23596113 | 4229660 | 1263664 | 8703782 | 81.6% | 75.5% |
| 15-2 | 30396229 | 27282337 | 17207022 | 3101308 | 921653 | 6537194 | 78.1% | 72.0% |

Duplication rates (Dup) were determined both by Position (Pos) and Sequence (Seq).
