# Supplementary material for: CRISPR-mediated HDAC2 disruption identifies two distinct classes of target genes in human cells
Source: PLoS One. 2017 Oct 5;12(10):e0185627. doi: 10.1371/journal.pone.0185627 (PMC5628847; doi:10.1371/journal.pone.0185627)
Supplement: S4 Table — (DOCX) [file pone.0185627.s010.docx]

**Somanath et al, Supplementary Information**

**S4 Table. qPCR assay primers**

| **Gene** | **Forward Primer** | **Reverse Primer** |
| --- | --- | --- |
| *HDAC1* | CGCCCTCACAAAGCCAATG | CTGCTTGCTGTACTCCGACA |
| *HDAC2* | GAGCTGTGAAGTTAAACCGACA | ACCGTCATTACACGATCTGTTG |
| *HDAC3* | TCTGGCTTCTGCTATGTCAACG | CCCGGTCAGTGAGGTAGAAAG |
| *BASP1* | AGGGGAACCCAAAAAGACTGA | GGTGTGGAACTAGGCGCTTC |
| *CDKN2C* | GGGGACCTAGAGCAACTTACT | CAGCGCAGTCCTTCCAAAT |
| *COL6A1* | ACAGTGACGAGGTGGAGATCA | GATAGCGCAGTCGGTGTAGG |
| *COL6A2* | GAACGGGACCGATGGACAG | CCCTTGGCCCCGATTTCTC |
| *LMNTD2* | TGATCCAGGAGTTGAAGGAACA | CCGTCACTACCTCCACACT |
| *NEFM* | AGGCCCTGACAGCCATTAC | CTCTTCGGCTTGGTCTGACTT |
| *PPP1R16A* | CCCTCCCAGTGTTGTCCTTC | ATGTTCCCGTCGGTGTTGAC |
| *RECQL4* | GTGGCCTCTAGGAAGAGCCT | AGCCAGGATCTAGGGAGCC |
| *CCT5* | TGGAACTGTCCAAGTCTCAGG | CATCGGCTATTCTGATTGGGTG |
| *SNX22* | CCTGTCCTGAGCTTCCATGTG | GGCTGGGCTTTATCTGGGC |
| *RPS6* | TGGACGATGAACGCAAACTTC | TTCGGACCACATAACCCTTCC |
| *TP53BP1* | ATGGACCCTACTGGAAGTCAG | TTTCTTTGTGCGTCTGGAGATT |
| *GAPDH* | GGAGCGAGATCCCTCCAAAAT | GGCTGTTGTCATACTTCTCATGG |
