# Supplementary material for: CRISPR-mediated HDAC2 disruption identifies two distinct classes of target genes in human cells
Source: PLoS One. 2017 Oct 5;12(10):e0185627. doi: 10.1371/journal.pone.0185627 (PMC5628847; doi:10.1371/journal.pone.0185627)
Supplement: S5 Table — (DOCX) [file pone.0185627.s011.docx]

**Somanath et al, Supplementary Information**

**S5 Table. ChIP-qPCR assay primers**

| **Gene** | **Forward Primer** | **Reverse Primer** |
| --- | --- | --- |
| *HDAC1* | GCTATAGGTGAGCCCAGGAG | GGTGCTCACCGTCGTAGTAG |
| *HDAC2* | GGGAAGGCTCGGTACCAC | CCCTAACAGGGCCTCTAACC |
| *HDAC3* | AGAAATAGGCCACGGTCTTG | CAGATACCGGGAGAGCTGAG |
| *BASP1* | ATTCTGTGTCCCGGTGCTAA | TCGGAACTTGGGAGAGGAAG |
| *CDKN2C* | TGGGTGAATGCCTTTTCTTC | TGACGACTCCACAGAGAGACA |
| *COL6A1* | AAGGAGGTGGTGACTCATGG | CCCAGCCAGAGTGAGAGC |
| *LMNTD2* | TCTCAAGCCAGAGACCCTTC | CCCCGAGACTACAGGTGTG |
| *PPP1R16A* | GACCTGCAGTGCTCTCTGTG | TTCAGGAGCACCTCCTCTTC |
| *RECQL4* | CGTCGTCCTGTAAAGGGAAC | GGAGATTCGCTGGACGAT |
| *CCT5* | TGGGTCCTACCATCTTCTCG | AGGGGGAGGTTTGAAGACAC |
| *SNX22* | GGACTGAGGAACCCAAGACA | TTGACGGAGCACTCACTGAT |
| *RPS6* | GCCAGTGGTGGTAGTCTGGT | CTCCCAAAGTGCTGGGATTA |
| *TP53BP1* | TCTGTCCCCTCCAATACTGC | TATCGTTGCCTCCATCTGTG |
