# Supplementary material for: CRISPR-mediated HDAC2 disruption identifies two distinct classes of target genes in human cells
Source: PLoS One. 2017 Oct 5;12(10):e0185627. doi: 10.1371/journal.pone.0185627 (PMC5628847; doi:10.1371/journal.pone.0185627)
Supplement: S8 Table — (DOCX) [file pone.0185627.s014.docx]

**Somanath et al, Supplemental Information**

**Table S8. ENCODE HDAC2 ChIP-seq peaks in Tier 1 cell lines present at validated HDAC2 targets.**

| **Gene** | **Cell Line** |
| --- | --- |
| *BASP1* | H1, MCF7, K562 |
| *COL6A1* | K562 |
| *COL6A2* | H1, K562 |
| *LMNTD2* | MCF7, K562 |
| *CDKN2C* | H1, MCF7, K562 |
| *PPP1R16A* | H1, MCF7, K562 |
| *NEFM* | H1, K562 |
| *CCT5* | H1, MCF7, K562 |
| *RECQL4* | H1, MCF7, K562 |
| *SNX22* | H1, MCF7, K562 |
| *RPS6* | H1, K562 |
| *TP53BP1* | H1, K562 |
